# Transcriptomic insights into the genetic basis of mammalian limb diversity

**DOI:** 10.1186/s12862-017-0902-6

**Published:** 2017-03-23

**Authors:** Jennifer A. Maier, Marcelo Rivas-Astroza, Jenny Deng, Anna Dowling, Paige Oboikovitz, Xiaoyi Cao, Richard R. Behringer, Chris J. Cretekos, John J. Rasweiler, Sheng Zhong, Karen E. Sears

**Affiliations:** 1 School of Integrative Biology, University of Illinois, 505 S Goodwin Avenue, Urbana, IL 61801 USA; 2 Institute for Genomic Biology, University of Illinois, 1206 W Gregory Drive, Urbana, IL 61801 USA; 3 Department of Bioengineering, University of California San Diego, 9500 Gilman Drive, La Jolla, CA 92093 USA; 4 Department of Genetics, University of Texas MD Anderson Cancer Center, 1515 Holcombe Boulevard, Houston, TX 77030 USA; 5 Department of Biological Sciences, Idaho State University, 921 South 8th Avenue, Pocatello, ID 83209 USA; 6 Department of Obstetrics and Gynecology, State University Downstate Medical Center, 450 Clarkson, Avenue, Brooklyn, NY 11203 USA

**Keywords:** Limb, Mammalian, Transcriptome, Diversification, Differential expression, Bat, Opossum, Pig, Mouse

## Abstract

**Background:**

From bat wings to whale flippers, limb diversification has been crucial to the evolutionary success of mammals. We performed the first transcriptome-wide study of limb development in multiple species to explore the hypothesis that mammalian limb diversification has proceeded through the differential expression of conserved shared genes, rather than by major changes to limb patterning. Specifically, we investigated the manner in which the expression of shared genes has evolved within and among mammalian species.

**Results:**

We assembled and compared transcriptomes of bat, mouse, opossum, and pig fore- and hind limbs at the ridge, bud, and paddle stages of development. Results suggest that gene expression patterns exhibit larger variation among species during later than earlier stages of limb development, while within species results are more mixed. Consistent with the former, results also suggest that genes expressed at later developmental stages tend to have a younger evolutionary age than genes expressed at earlier stages. A suite of key limb-patterning genes was identified as being differentially expressed among the homologous limbs of all species. However, only a small subset of shared genes is differentially expressed in the fore- and hind limbs of all examined species. Similarly, a small subset of shared genes is differentially expressed within the fore- and hind limb of a single species and among the forelimbs of different species.

**Conclusions:**

Taken together, results of this study do not support the existence of a phylotypic period of limb development ending at chondrogenesis, but do support the hypothesis that the hierarchical nature of development translates into increasing variation among species as development progresses.

**Electronic supplementary material:**

The online version of this article (doi:10.1186/s12862-017-0902-6) contains supplementary material, which is available to authorized users.

## Background

Drivers of morphological diversification include the evolutionary birth of new genes and changes in the regulation of orthologous genes. For some time, many biologists have argued that modifications in gene regulation have triggered many if not most of the evolutionary changes in morphology that characterize the history of life [1–12]. However, the specific changes in gene regulation and expression that have driven morphological diversity remain unknown for most systems. This study employs a comprehensive, multi-species comparison of the gene expression in developing mammalian limbs to investigate how the expression of the highly conserved genes governing organ morphogenesis have been modified during the diversification of limb morphology within and among species.

Mammalian limbs represent an exceptional case study for investigating the evolution of gene expression, as they have undergone extensive evolutionary diversification [13] and represent a well-characterized model system for development [14, 15]. Furthermore, from the wings of bats to the flippers of whales to the one-toed hooves of horses, mammalian limb diversification has been crucial to the success of the group. For example, through the morphological diversification of their limbs, mammals were able to infiltrate almost every habitat in the world, and exhibit a wide-range of feeding and social behaviors [13]. To date, studies of mammalian limb evolution and development has been limited mostly to investigations of the role of candidate genes (e.g., bats [3, 11, 12, 16–19], whales [4], opossums [20–23], non-cetacean artiodactyls [8], jerboas [8]), or, in a couple of cases, transcriptomes [24–27], in individual species. From these studies we have learned that mammalian limb diversification has proceeded not by major changes to limb structure (e.g., complete loss or gain of entire segments), but by the modification of segments inherited from their generalized, pentadactyl ancestor [28, 29]. Accordingly, these studies of the candidate genes and transcriptomes of isolated species studies suggest that mammalian limb diversification has likely been driven largely by the differential expression of conserved genes shared by all mammals [2–4, 8, 11, 30–32].

While studies of the candidate genes and transcriptomes of isolated species have significantly advanced our understanding of mammalian limb evolution, and morphological evolution in general, there are several outstanding questions concerning limb developmental evolution that have proven difficult to answer with these approaches. For example, we do not yet have a comprehensive view of when, developmentally, the expression pattern of shared genes diverges among the fore- and hind limbs of a single species, or among the limbs of different species. Development is a hierarchical process that builds on itself as time progresses. Because of this, some biologists have proposed that the developmental processes mediating earlier developmental events (e.g., initial specification of organ fields) might be less variable than those governing later events (e.g., organ specialization) [33–40]. The earlier stages of limb development also coincide with the proposed phylotypic period for vertebrates, which is the period in which vertebrate species most closely resemble each other [41, 42]. The phylotypic period has been proposed to encompass the initiation and early development of the limb bud prior to the onset of chondrogenesis [37, 43–45]. Strong inductive signaling between different parts of the embryo has been proposed to characterize the phylotypic period, and result in the conservation of this stage across vertebrates [37, 41, 46]. Evidence for a phylotypic period has recently been observed in other systems, including plants [47], flies [48], zebrafish [49], nematodes [50], and across several vertebrates [51]. Our first working hypothesis is therefore that the expression pattern of shared genes tends to diverge later (i.e., at or after the onset of chondrogenesis) rather than earlier (i.e., before the onset of chondrogenesis) in limb development.

We also do not have a complete picture of which shared genes and gene pathways differ the most in their expression among the fore- and hind limbs of a single species and among the limbs of different species. Candidate gene approaches have identified differences in the expression of major limb patterning genes (e.g., *Shh*, *Fgf*’s, *Hox* genes, *Bmp*’s, etc.) across mammalian species [3, 4, 8, 12, 16, 17, 19]. However, these studies were not comprehensive in their gene coverage. Our second working hypothesis is that the expression patterns of several, major limb patterning genes significantly differ between the fore- and hind limbs of a single mammalian species, and among the limbs of different mammalian species.

Finally, we do not know the degree to which different species share a common pattern of gene expression divergence (e.g., timing, genes involved, etc.) between their fore- and hind limbs. Similarly, we do not know the degree of similarity between the patterns of gene expression divergence within (e.g., between the fore- and hind limb of a single species) and among (e.g., between the forelimbs of two different species) species. If certain aspects of the genetic basis of limb development are more evolvable than others, then we might expect the pattern of gene expression divergence to be conserved within species, and within and among species. Our third working hypothesis is therefore that the same genes are differentially expressed in the fore- and hind limbs of several species, and among the limbs of species.

**Fig. 1:**
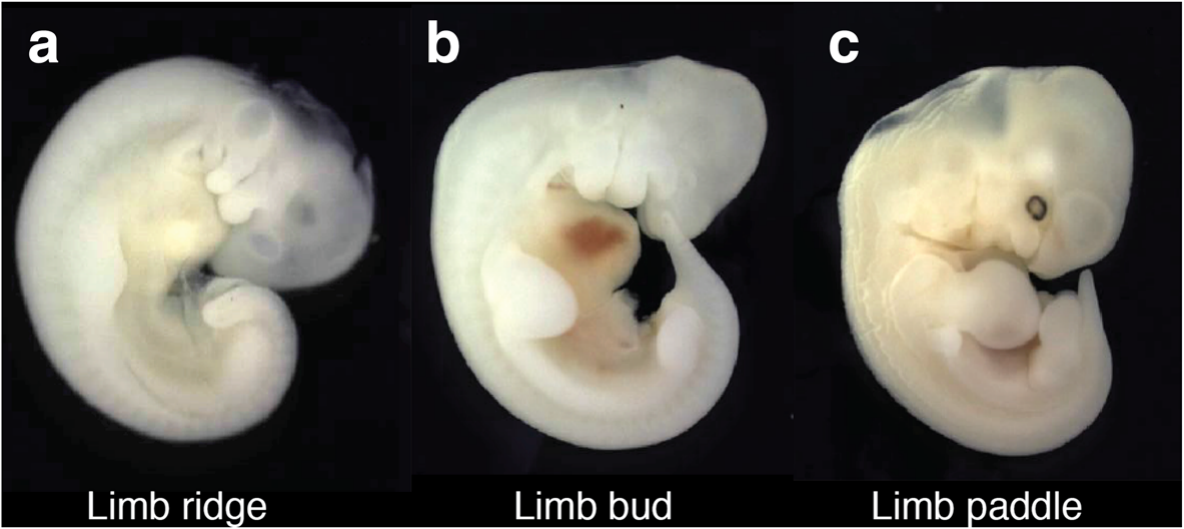
Representative stages of limb development used in this study. This study assembled the transcriptomes of four species (bat, mouse, opossum, and pig) from three stages of limb development, namely the ridge (**a**), bud (**b**), and paddle (**c**) stages. The embryos shown here are from *Erophylla sezekorni* (Buffy Flower Bat)

To test these hypotheses we performed the first comprehensive, multi-species comparison of the transcriptomes of developing mammalian limbs. We compared the overall patterns of gene expression in the limbs of four mammals that are taxonomically diverse and represent extremes of mammalian limb development and adult limb structure, namely bats (*Carollia perspicillata*), pigs (*Sus scrofa*), mice (*Mus musculus*), and opossums (*Monodelphis domestica*). These species include a generalized, pentadactyl mammal (mouse), a representative of the only mammalian group to evolve a wing capable of powered flight (bat), a marsupial whose hind limb development lags significantly behind forelimb development relative to eutherian mammals (opossum) [52], and a mammal that displays digit reduction (pig). We performed RNA-Seq on the developing fore- and hindlimbs of these four mammalian species [53]. We then quantified the transcriptomes of the fore- and hind limbs of these species at the ridge, bud, and paddle stages of development (Fig. 1) [54–56]. The limb first begins to grow out from the flank during the ridge stage, becomes a semicircle that is as long as it is wide during the bud stage, and forms a hand-plate during the paddle stage. Condensation of the limb cartilaginous elements of the limb begins between the bud and paddle stages [23, 56–58]. We then used the results to determine the pattern of transcriptomic divergence among the forelimbs of different species, the hind limbs of different species, and the fore- and hind limbs of a single species. For several genes, we also assay their spatial expression domains using whole mount *in situ* hybridization.

Results of the among-species analyses of this study are consistent with the pattern of gene expression during initial limb outgrowth being conserved among mammals relative to subsequent stages of limb development, while within-species results are more mixed. In regard to the former, results further suggest that the development of the limbs of different species likely begins to diverge before the onset of cartilage condensation. Results also suggest that the expression patterns of the limb development genes *Hand1*, *Isl1*, *Myog*, *Pax1*, *Tbx4*, *Tbx5*, and *Tnnt2* significantly differ between the fore- and hind limbs of all mammalian species. This study also identified several genes with known roles in limb development that display greatly divergent expression in the fore- and hind limbs of most species and among the limbs of all species (e.g., *Col2a1*, *Hoxa13*, *Mecom*, *Pitx1*, *Rarb*, *Tbx4*, *Wnt5a*, *Zbtb16*). However, results suggest that, on the whole, the genes that are differentially expressed in fore- and hind limbs differ from species to species, and that the genes that are that are differentially expressed within (e.g., between the fore- and hind limb of a single species) and among (e.g., between the forelimbs of two different species) species are generally distinct. This study therefore identifies a small subset of genes with possible roles in the generation of the distinct morphologies of the fore- and hind limbs, and a small but different subset with possible roles in the evolutionary divergence of limb morphology across species. This result, combined with the observed differences in the overall patterns of among- and within- species variation, suggests that the processes controlling within- and among- species variation may differ. This study therefore provides tangible insights into the pattern of gene expression divergence during the specialization of mammalian limbs, and the coupled achievement of mammalian diversity.

## Results

**Fig. 2:**
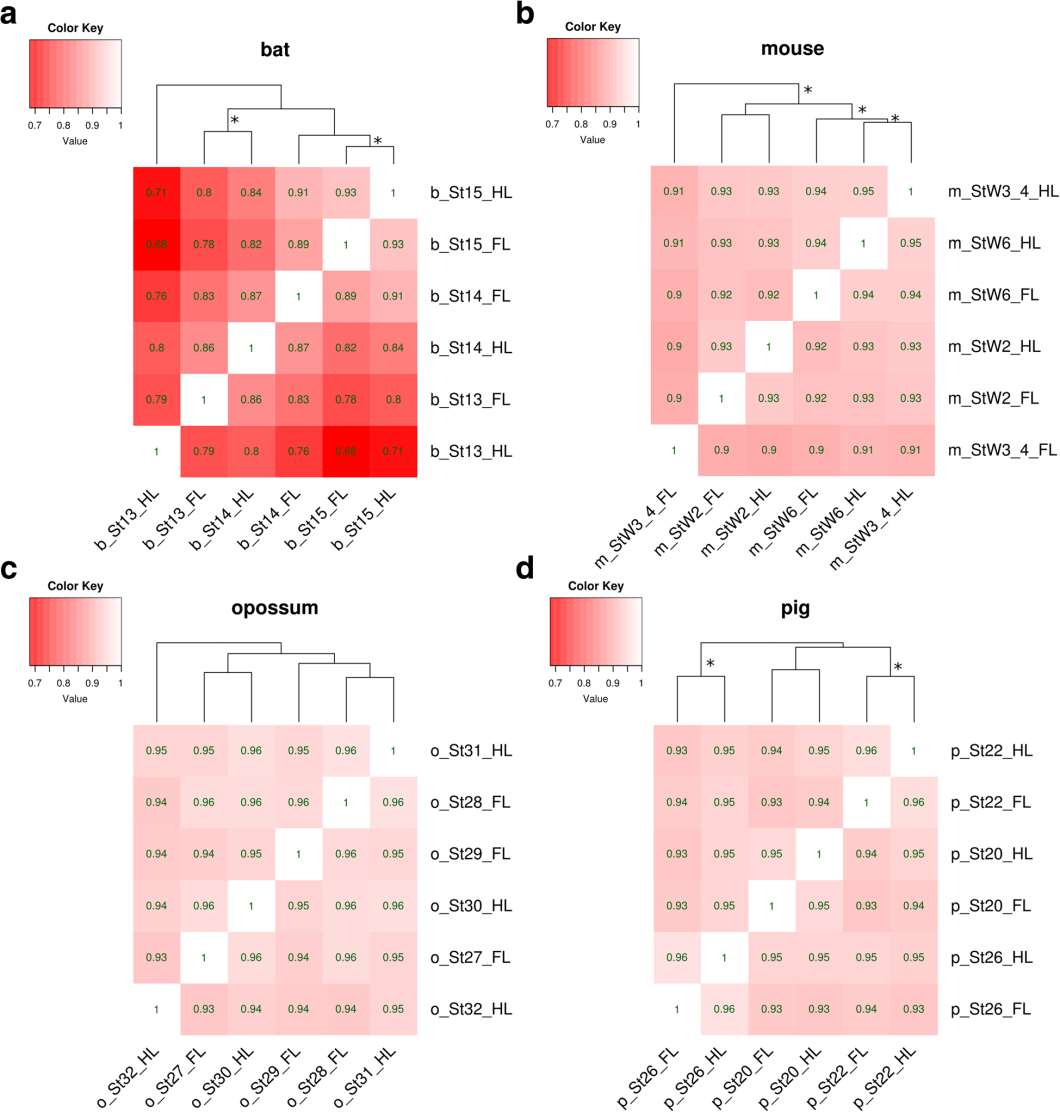
Similarity of gene expression profiles *within* species. Heat-maps of the gene expression profiles for the fore- and hind limbs of all developmental stages for bat (**a**), mouse (**b**), opossum (**c**), and pig (**d**). FL = forelimb, HL = hind limb. The stages for each species are as follows: b_St13 = bat ridge, b_St14 = bat bud, b_St15 = bat paddle, m_StW2 = mouse ridge, m_StW3_4 = mouse bud, mStW6 = mouse paddle, o_St27 = opossum ridge, o_St28 = opossum bud, o_St29 = opossum paddle, o_St30 = opossum ridge, o_St31 = opossum bud, o_St32 = opossum paddle, p_St20 = pig ridge, p_St22 = pig bud, p_St26 = pig paddle. Branches with statistically significant clustered sub-branches (p-value < =0.05) are indicated with asterisks (*)

*Timing of the divergence of gene expression of shared genes within species (fore-* vs. *hind limb)* – Limbs at the ridge, bud, and paddle stages (Fig. 1) were removed from each species and total RNA purified (see Materials and Methods and [53] for more details). We used the Illumina TruSeq RNA Sample Preparation Kit to generate libraries. Once libraries were generated and sequenced, data were quality controlled before analysis (See Additional file 1 and Additional file 2: Figures S1 & Additional file 3: Figure S2 for full details). We generated heat-maps to visualize the similarity of gene expression profiles for the fore- and hind limbs of all developmental stages for a given species (Fig. 2), using the pvclust R package to determine the significance of the clusters [59] and (Additional file 4: Figure S3). In bats (Fig. 2a), the fore- and hind limbs cluster together at the paddle stage of development, with the forelimb bud falling at the next step out. The forelimb ridge and hind limb bud also cluster together, with the hind limb ridge as the outlier for all limbs and stages. Of these, the clusters of the paddle stage fore- and hind limbs and of the forelimb ridge and hind limb bud are significant (*P*-value < 0.05) (Additional file 4: Figure S3A). In mouse, the hind limb bud and paddle cluster together with the forelimb paddle as the next step out, and the fore- and hind limb ridges cluster together (Fig. 2b). The mouse forelimb bud is the outlier. The cluster of the fore- and hind limb ridge, fore- and hind limb paddle, and hind limb bud stages is significant (Additional file 3: Figure S2B). In opossum, the fore- and hind limb buds cluster together and the fore- and hind limb ridges cluster together, with the forelimb paddle clustering with the bud samples and the hind limb paddle falling as an outgroup to all other samples (Fig. 2c). None of these clusters are significant (Additional file 4: Figure S3C). Finally, in pig the fore- and hind limbs cluster together at every developmental stage, with the ridge and bud stages clustering together to the exclusion of the paddle samples (Fig. 2d). Of these, the bud and paddle clusters of the fore- and hind limbs are significant (Additional file 4: Figure S3D). Thus the overall results of the clustering are mixed. The ridge staged fore- and hind limbs cluster together in three of the four species, and the bud and paddle staged limbs cluster in two. However, only four of these clusters are statistically supported (one ridge, one bud, and two paddle).

**Fig. 3:**
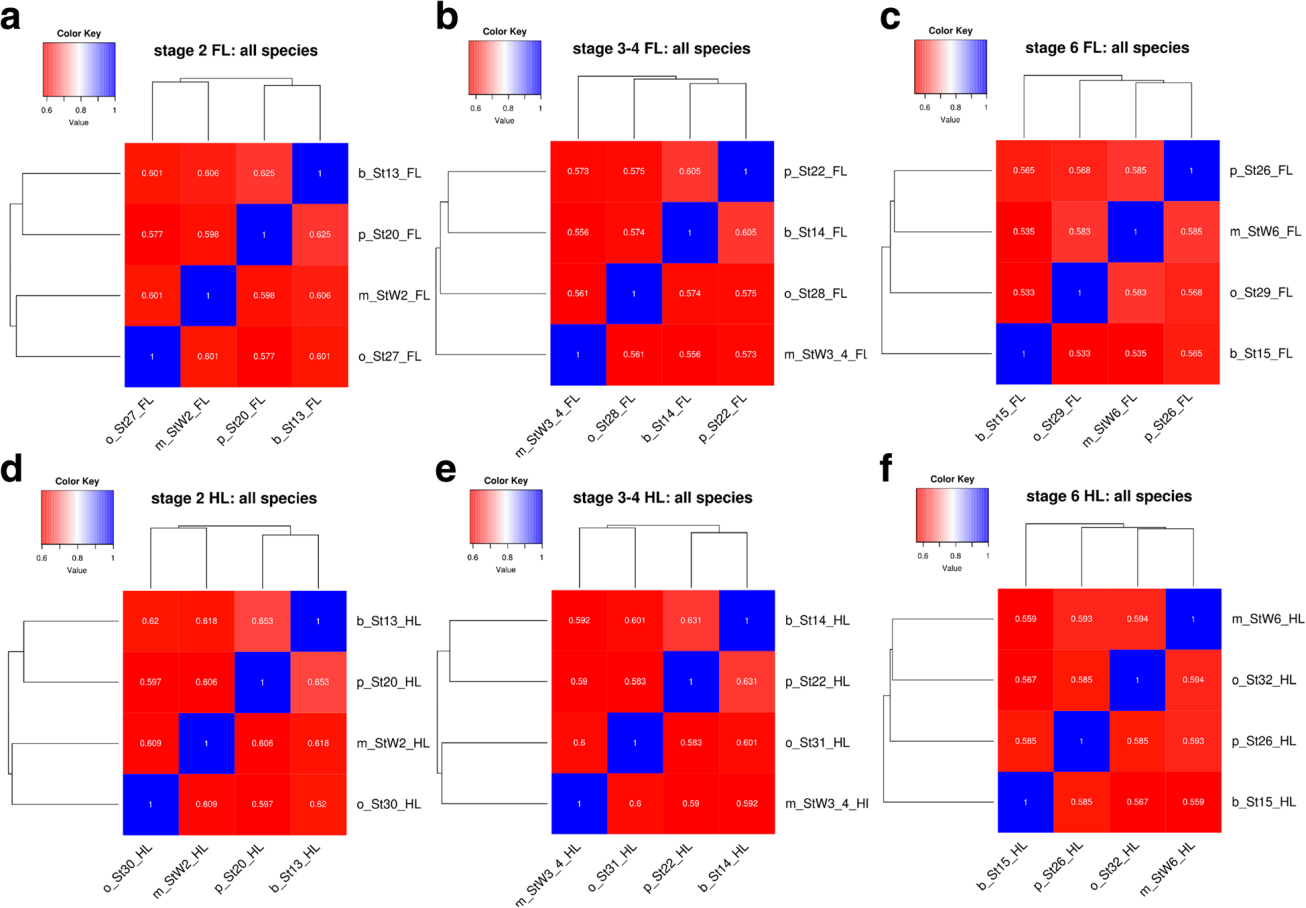
Similarity of gene expression profiles *among* species. Pairwise Spearman coefficient values for forelimbs at the (**a**), bud (**b**), paddle (**c**) stages for all species, and for the hind limbs of the ridge (**d**), bud (**e**), paddle (**f**) stages for all species. M. = mouse, O. = opossum. Abbreviations for stages and species are as in the legend for Fig. 2. In each label for the heat map, Stage 2 corresponds to ridge, Stage 3–4 to bud, and Stage 6 to paddle

*Timing of the divergence of shared gene expression among species (fore-* vs. *forelimb, hind* vs. *hind limb)* - Pairwise species coefficients were generated for 6,583 orthologs in the 4 species to measure between-species gene expression conservation at each developmental stage (See Additional file 1 for more details). The results of these correlations were used to generate heat-maps to visualize the similarity of gene expression profiles for the forelimbs of all developmental stages for all species, and for the hind limbs (Fig. 3). All species were positively correlated (Spearman coefficient >0.5), however, none of these correlations were significant at the *P*-value < 0.05 level (Additional file 5: Figure S4). For the ridge stage of both the fore- and hind limb, and the bud stage of the hind limb, bat and pig cluster together, and mouse and opossum cluster together (Fig. 3a, d, e). For pig and bat, this clustering pattern matches the phylogenetic relationships between these species [60]. At the bud stage of forelimb development (Fig. 3b), bat and pig clusters remain clustered together, followed by opossum, and then mouse. Finally, for the paddle stage of development, pig and mouse cluster together, followed by opossum and then bat for the forelimb (Fig. 3c). In the hind limb, mouse and opossum cluster together, followed by pig and then bat (Fig. 3f). Therefore bat, with its highly divergent limb morphology, is an outlier for the paddle stage of development for both the fore- and hind limb.

**Fig. 4:**
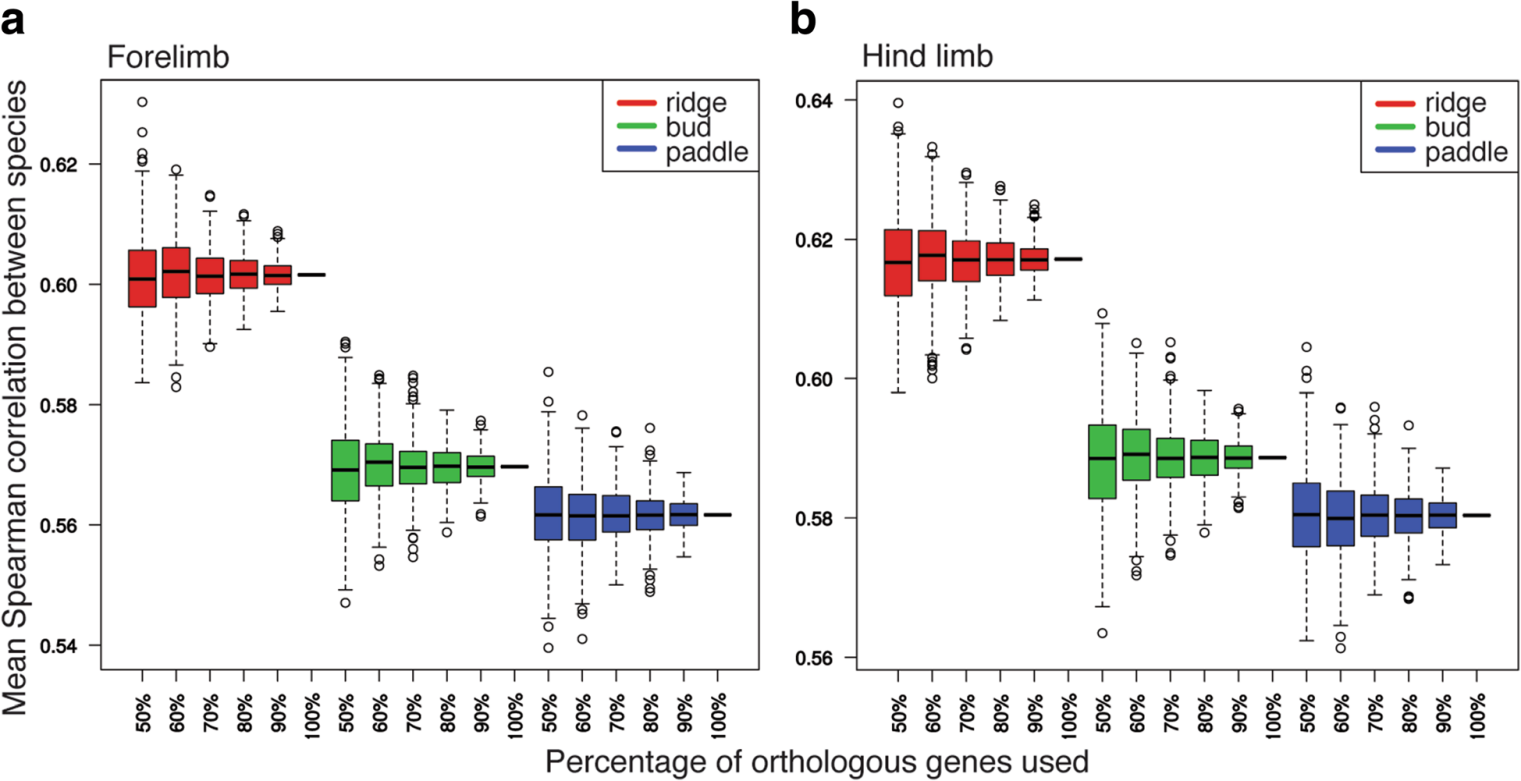
Conservation of gene expression profiles. The conservation of the gene expression profiles of all study species (bat, mouse, opossum, and pig) across embryonic development of the fore- (**a**) and hind limb (**b**), as determined by the mean of all species pairwise Spearman coefficients (X axis). Results for the ridge stage are shown in red, for the bud stage in green, and for the paddle stage in blue. To test the robustness of differences in Spearman coefficients with respect to the gene selection, we randomly sub-sampled gene sets at intensities ranging from 50 to 100% of all genes (Y axis). In the box plots, the bottom and top of the boxes represent the first and third quartiles of the data, and the line through the middle of the box the second quartile (median). The whiskers range from the 2^nd^ to 98^th^ percentiles, and the open circles depict the outliers to these percentiles

We also calculated the conservation of the gene expression profiles of bat, mouse, opossum, and pig across embryonic limb development, using the mean of all species pairwise Spearman coefficients. All resulting Spearman coefficients are positive and > 0.50, suggesting that the orthologous genes might perform similar functions between species (Figs. 4a, 4b). In both the fore- and hind limb, the degree of gene expression conservation decreases from the ridge (forelimb = 0.6011, hind limb = 0.6170) to bud (forelimb = 0.5697, hind limb = 0.5882) to paddle (forelimb = 0.5613, hind limb = 0.5799) stage. When all the genes are sampled, the distributions of gene expression conservation levels between the ridge, bud, and paddle stages are significantly different (*T*-test, *P*-value < 0.05*). To test the robustness of this difference with respect to the selection of orthologous genes, we randomly sub-sampled 500 sets of orthologous genes at all developmental stages at intensities ranging from 50 to 100% of all orthologous genes. According to the resulting distributions (Fig. 4), only 70% of the genes need to be sampled to find a statistically significant difference (non-overlapping 95% confidence intervals) between the ridge and bud stages of forelimb and of hind limb development. However, the significances of the differences between the bud and paddle stages of the forelimb and of the hind limb are more dependent on the genes that are sampled. The distributions indicate that over 90% of orthologous genes must be sampled to find significant differences (non-overlapping 95% confidence intervals) between these stages.

**Fig. 5:**
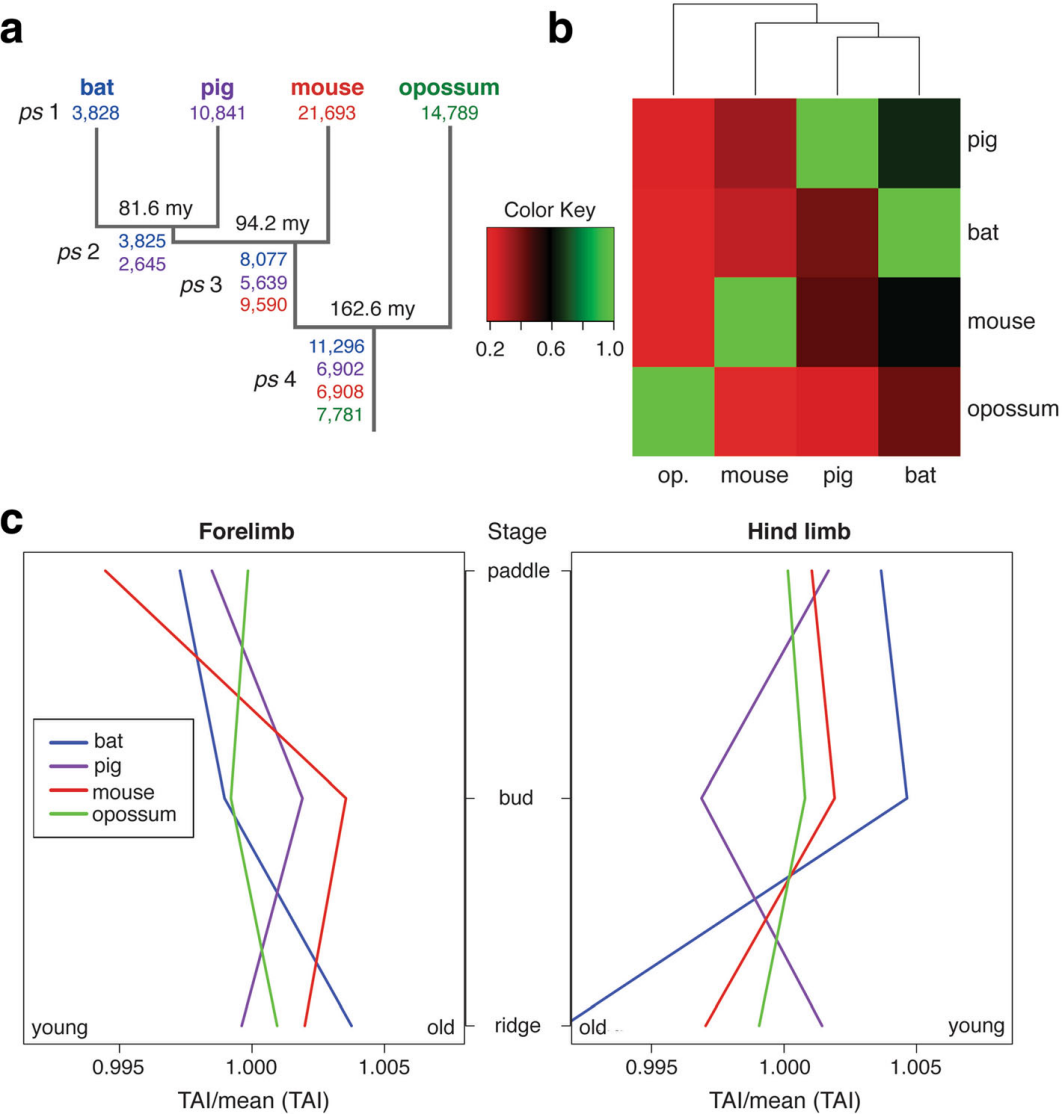
Evolutionary Age. The evolutionary ages of the genes of each species in this study are shown in (**a**). Genes were assigned to phylostratums (*ps*) based on their degree of conservation. At one end of the spectrum, genes that are specific to a single species (i.e., they do not have homologues in other species) were assigned to *ps* 1, and at the other end genes that are common to all study species were assigned to *ps* 4. *Ps* 2 and *ps* 3 correspond to the branching points of the phylogenetic tree between *ps* 1 and *ps* 4. Genes in *ps 1*, *ps 2*, *ps 3*, and *ps 4* were assigned evolutionary ages of 1, 2, 3, and 4, respectively. A hierarchical clustering analysis based on the pair-wise number of homologue genes (**b**) exactly mirrors the phylogeny of the study species. Results shown in (**a**) were used to calculate the transcriptome age indices (*TAIs*) of each species across development (**c**) for the fore- (left) and hind (right) limbs. Smaller *TAIs* correspond to younger ages, and larger *TAIs* older ages. Bat is shown in blue, pig in purple, mouse in red, and opossum in green

To further investigate the pattern of gene expression divergence among species, we calculated the evolutionary age of the genes of each species in our study. For this analysis, evolutionary age is defined by the phylogenetic origin of a set of genes, or its phylostratum. Gene sets with lower phylostrata have a younger evolutionary age, and vice versa. We found that bat, pig, mouse, and opossum have 3,828, 10,841, 21,693, and 14,789 genes, respectively, that are specific to a single species (i.e., they do not have a homologue in any other species) (Fig. 5a). For this, all genes (protein-coding and non-protein coding) provided by the ENSEMBL annotations were used. These genes were assigned to phylostratum (*ps*) 1, and their evolutionary age was assigned a value of 1. On the other end of the spectrum, the number of genes that are common among all four species (*ps* 4) ranges from 6,902 (pig) to 11,296 (bat). These genes were assigned an evolutionary age of 4. *Ps* 2 and *ps* 3 fall between the youngest (*ps* 1) and oldest (*ps* 4) genes, and correspond to the branching points of the phylogenetic tree (Fig. 5a). The age of each set of genes was assigned in accordance to its *ps*. To test if the assignment of homologous genes between any two species was coherent with the expected phylogeny, we performed a hierarchical clustering analysis based on the pair-wise number of homologous genes. We found that the clustering exactly mirrors the phylogeny of the study species (Fig. 5b), supporting the method we used to call homologous genes.

We then calculated the specificity of the transcriptome of each species across development using the transcriptome age index (*TAIs*) (Fig. 5c). For the forelimbs of mouse and pig, we found that younger genes dominate the transcriptomes of the ridge and paddle stages, while older genes dominate the transcriptome of the bud stage. The forelimb of opossum showed the opposite trend, with older genes dominating the transcriptomes of the ridge and paddle stages and younger genes the transcriptome of the bud stage. Bat demonstrated yet another pattern, with younger genes increasingly dominating the transcriptome as limb development progressed. When we analyzed the statistical significance of the *TAIs* using VTAI (variance of TAIs) as a test statistic, [47] we found that the bat, mouse, and pig forelimb trends are highly significant (*P*-values = 1.11e-4, 1.36e-4, and 3.12e-4, respectively), whereas the opossum forelimb trend is not (*P*-value = 0.256) (Additional file 6: Figure S5). The same analysis was done for TAIs and VTAI in the hind limbs. In the hind limb, the bat, mouse, and opossum transcriptomes are dominated by older genes at the ridge and paddle stages, and by younger genes at the bud stage. The pig hind limb shows the opposite trend, with the bud stage being dominated by older genes, and the ridge and paddle stages by younger genes. The hind limb trends for bat, mouse, and pig are highly significant (*P*-values = 5.08e-4, 2.29e-10, and 3.12e-4, respectively) while that of opossum is not significant (*P*-value = 0.151) (Additional file 7: Figure S6). The lack of statistical significance of opossum results may stem from the reduced number of evolutionary ages that could be assigned to its genes (*ps* 1 and *ps* 4). Overall, the genes that are expressed at the paddle stage are younger than those expressed at the ridge stage for the fore- and hind limbs of all species (Fig. 5c).

*Similarity of gene expression patterns within (fore-* vs. *hind limbs) different species* – We next determined the degree to which patterns of fore- and hind limb gene expression divergence are similar in the different study species. We found that 4.5% of genes (*N* = 56) exhibit divergent expression (75^th^ percentile and above) in the fore- and hind limbs of all species during the ridge stage of development, 4.3% of genes (*N* = 51) exhibit divergent expression during the bud stage, and 8.4% of genes (*N* = 109) exhibit divergent expression during the paddle stage. These include genes with well-established differences in expression in the fore- and hind limbs (e.g., *Tbx4*, *Tbx5*) as well as additional genes with known roles in limb development (e.g., *Grem1*, *Wnt5a*) (Additional file 8: Table S1) [15, 61–64]. Of note, these genes do not include *Pitx1*, a gene known to play a fundamental role in the establishment of fore- and hind limb identity in mammals [15]. The reason for this is that *Pitx1* is not present in the pig genome (Sscrofa10.2) we used for our alignment. Therefore, although our results suggest that *Pitx1* exhibits divergent expression in the fore- and hind limbs of bat, opossum, and mouse, we have no data on *Pitx1* expression in pig. In total, seven genes are divergently expressed in the fore- and hind limbs of all species at all stages (*Hand1*, *Isl1*, *Myog*, *Pax1*, *Tbx4*, *Tbx5*, *Tnnt2*). This is consistent with literature of some of these genes being differentially expressed in the fore- and hind limb (see Discussion).

*Similarity of gene expression patterns among (fore-* vs. *fore- limb, hind* vs. *hind limb) different species* – We also compared the genes that display the most divergent expression (75^th^ percentile and above) among the limbs of species, and found that several have known roles in limb development. At the forelimb ridge, bud, and paddle stages, 12, 7, and 7 divergently expressed genes have known roles in limb development, respectively (Additional file 9: Table S2). These genes include members of the *HoxD* family and related genes (*Evx2*, *Lnp*), as well as the signaling factor genes *Fgf8* and *Shh*. In the hind limb, the ridge, bud, and paddle stages contain 17, 19, and 18 divergently expressed genes with known roles in limb development, respectively (Additional file 9: Table S2). These genes include additional members of the *Hox* family and related genes (*Hoxa13*, *Hoxd9*, *Hoxd11*, *Hoxd13*, *Lnp*), as well as *Fgf8*. Two limb genes (*Hoxa13* and *Med1*) are divergently expressed during all three stages of forelimb development, while nine (*Ctnnb1*, *Fbn2*, *Hoxd11*, *Med1*, *Pitx1*, *Prrx1*, *Rarb*, *Tbx4*, and *Twist1*) are divergently expressed at all three stages of hind limb development. Thus, many of the most divergently expressed genes at all stages in the fore- and hindlimbs in our species under study are known to play a role in limb development.

*Confirmation of gene expression patterns*: To further examine the expression of select genes from the *Hoxa* and *Hoxd* clusters (i.e., *Hoxd12*, *Hoxd13*, *Evx2*, *Hoxa13*), we cloned the coding sequences for bat and opossum and generated species-specific probes for WISH. We focused on the *Hoxa* and *Hoxd* clusters because of their well-established role in limb outgrowth and patterning in mice [65, 66]. In general, WISH results were consistent with those of RNA-Seq.

**Fig. 6:**
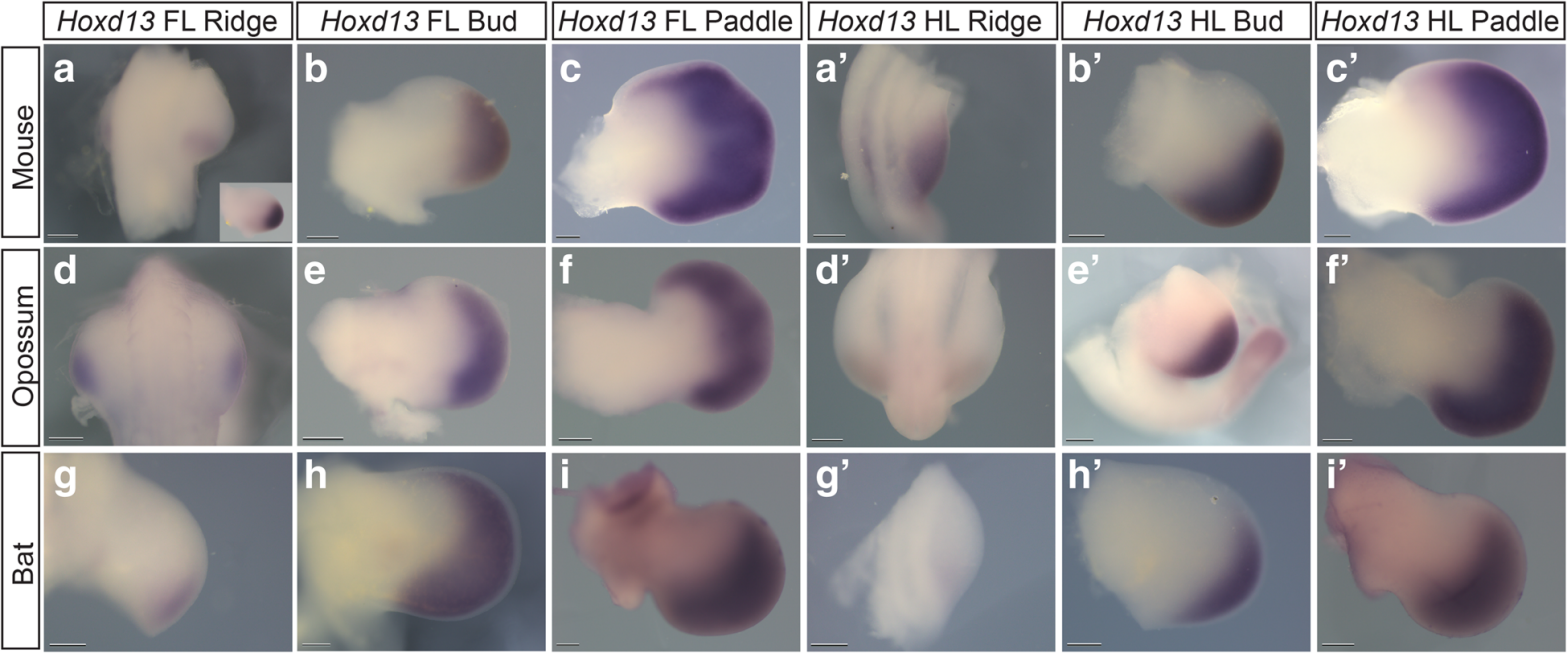
*Hoxd13* Expression in Limb Stages of Three Species. **a**-**c**: Mouse forelimb *Hoxd13* in ridge, bud, and paddle, respectively. **a**’-**c**’: Mouse hindlimb *Hoxd13* in ridge, bud, and paddle. **d**-**f** and **d**’-**f**’: Opossum *Hoxd13* in forelimb and hindlimb ridge, bud, and paddle, respectively. **g**-**i** and **g**’-**i**’: Bat *Hoxd13* in forelimb and hindlimb ridge, bud, and paddle. Purple staining denotes *Hoxd13* expression boundary. In bat forelimb ridge (**g**) the posterior limb was damaged in processing, though the area of *Hoxd13* expression is intact. The inset in **a** is an earlier bud stage between that of ridge and image shown in **b**. Scale bars = 0.2 mm

**Fig. 7:**
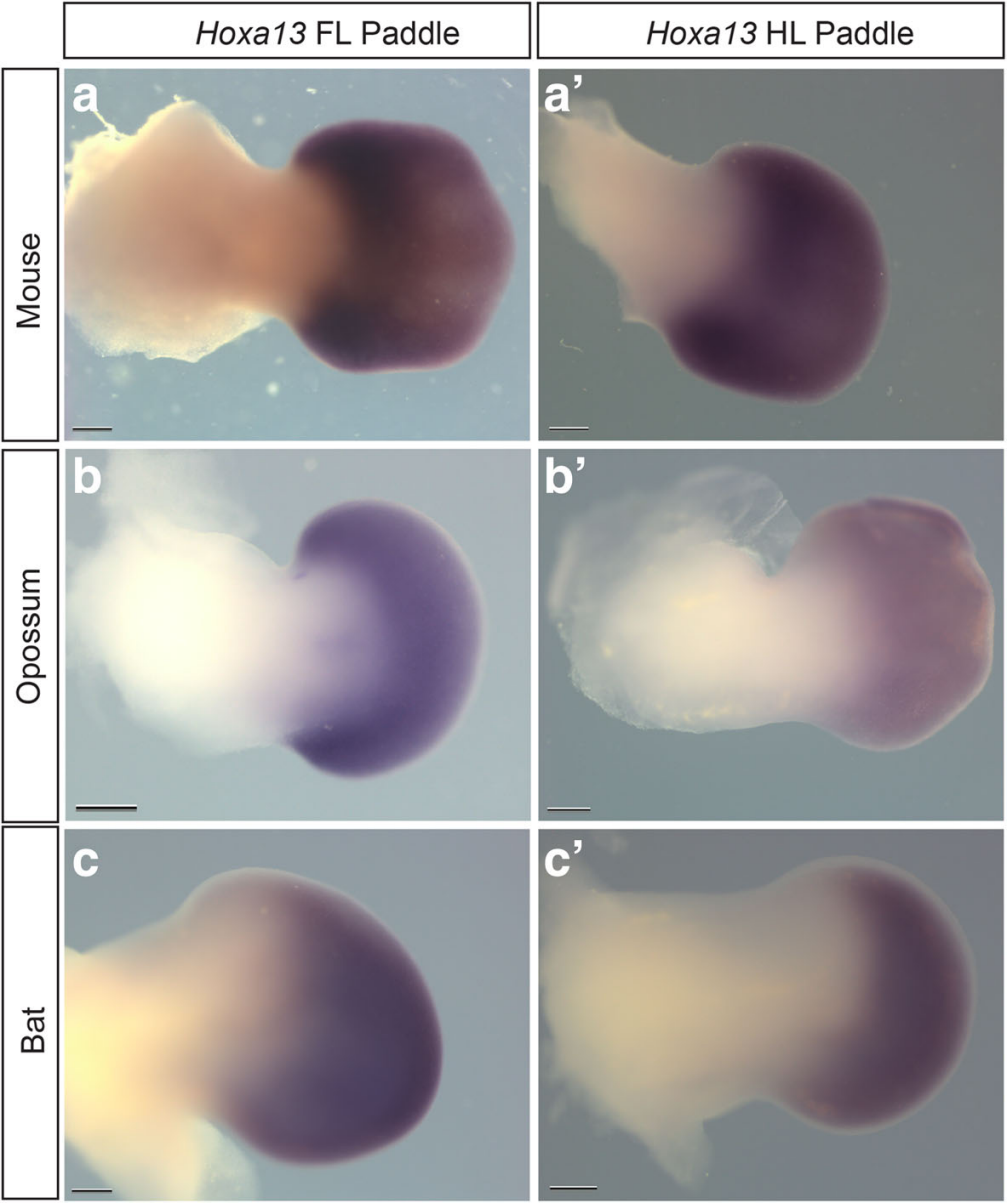
*Hoxa13* Expression in Stages of Mouse, Opossum, and Bat Paddle Stages. **a**, **a**’: Fore- and hindlimb of mouse, respectively. **b**, **b**’: Opossum, **c**, **c**’: Bat

In all species, the *Hoxd13* expression domain is confined posteriorly in the fore- and hind limbs before expanding anteriorly. However, the timing and degree of this expansion differs. The degree of anterior-expansion of the *Hoxd13* expression domain is greater and more symmetrical in the ridge- and bud-staged forelimbs of opossum (Fig. 6d, e) relative to mouse (Fig. 6a, b), but roughly comparable in paddle-staged opossum (Fig. 6f) and mouse (Fig. 6c) forelimbs. In contrast, the anterior-most boundary of the *Hoxd13* expression domain is located more posteriorly in the forelimb paddles of bat (Fig. 6i) relative to mouse (Fig. 6c) and opossum (Fig. 6f). This pattern of early, expanded anterior expression in opossum (not shown) and relatively anteriorly restricted expression in bat are also observed for *Hoxa13* (paddle stage shown in Fig. 7). The bat forelimb bud (Fig. 6h) also has a proximodistally wider *Hoxd13* expression domain than that of the bat hind limb bud (Fig. 6h’). *Hoxd12* (Additional file 10: Figure S10) had similar expression domains in bats, and opossums for stages examined and were generally consistent with published results for mouse *Hoxd12* [67–69].

**Fig. 8:**
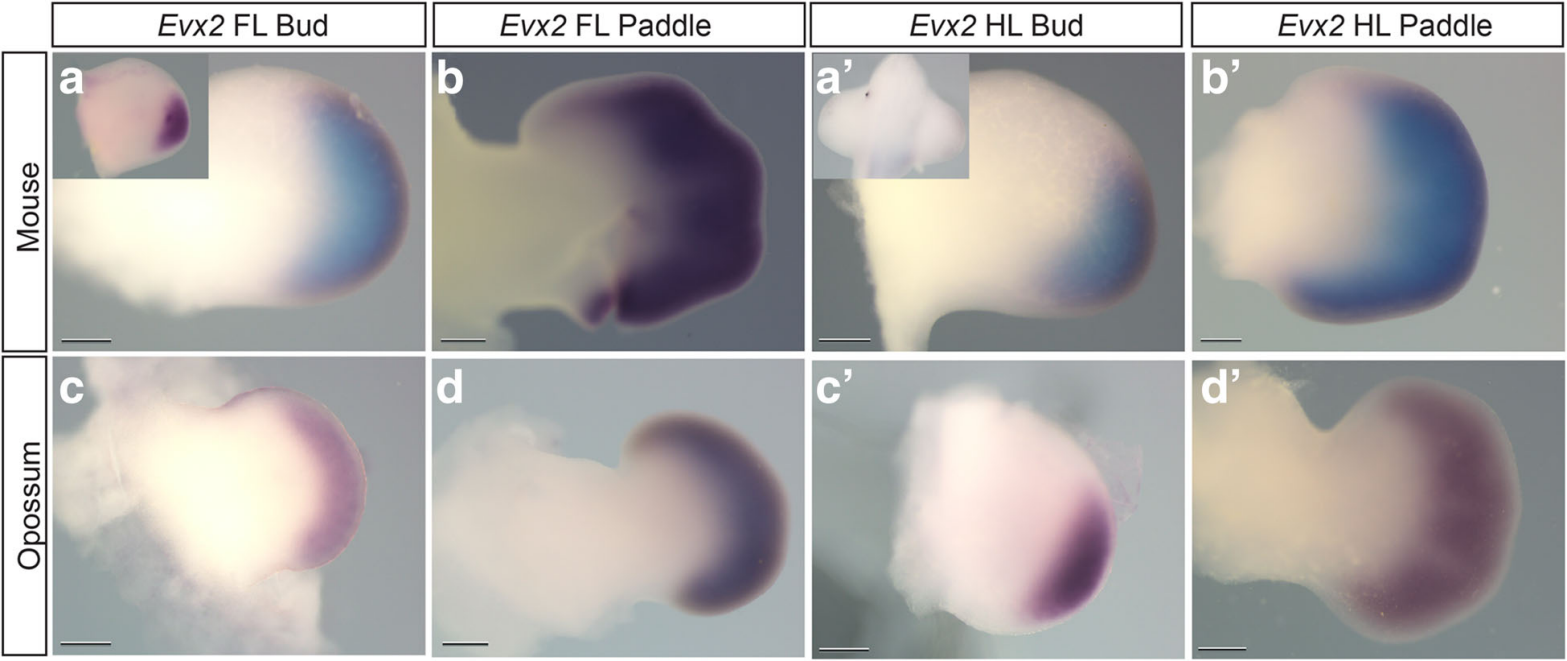
*Evx2* Expression in Limb Stages of Mice and Opossums. **a**-**b**, **a**’-**b**’: Mouse *Evx2* in forelimb and hindlimb of the bud and paddle, respectively. **c**-**d**, **c**’-**d**’: Opossum *Evx2* in forelimb and hindlimb of bud and paddle, respectively. The insets in **a** and **a**’ are mouse *Evx2* in earlier fore- and hindlimb buds, respectively. Scale bars = 0.2 mm

The expression domain of *Evx2*, a gene that shares regulation with the *Hoxd* cluster [70–72], is also initially expanded anteriorly in opossum forelimb buds and paddles relative to those of mice (Fig. 8). In mouse, forelimb expression appears in the posterior limb during the early bud stage (Fig. 8a, inset), expands anteriorly during the later bud stage (Fig. 8a), and by the paddle (Fig. 8b) is broadly present across the limb. In contrast, *Evx2* is not expressed at ridge (not shown) and early bud stages (Fig. 8a’ inset) of the mouse hind limb, before it appears in the posterior of the limb later in the bud stage (Fig. 8a’) and expands anteriorly in the paddle stage (Fig. 8b’). In opossum, *Evx2* is expressed more broadly along the posterior-anterior axis in the fore- (Fig. 8c) than hind limb (Fig. 8c’) bud. Similar to *Hoxd13*, the opossum *Evx2* expression domain is expanded anteriorly and has a more symmetrical domain in the forelimb bud relative to mouse. Furthermore, *Evx2* expression is not present in the early hind limb bud stages in mouse (see Fig. 8a’ inset) while it is robustly expressed at this stage in opossum (Fig. 8c’). For additional replicates of *Hoxa13*, *Evx2*, and *Hoxd13* WISH, see Additional file 11: Figure S7, Additional file 12: Figure S8, Additional file 13: Figure S9.

*Similarity of gene expression patterns within (fore-* vs. *hind limb) and among (fore-* vs. *fore- limb, hind* vs. *hind limb) species* – We also compared the genes that display the most divergent expression between the fore- and hind limbs of a single species, and among the limbs of species (75^th^ percentile and above). We found that 2.02 to 6.87% of genes exhibit greatly divergent expression in the fore- and hind limb of a single species and among the forelimbs of all species (average = 4.14%). Similarly, 1.79 to 5.35% of genes exhibit greatly divergent expression in the fore- and hind limb of a single species and among the hind limbs of all species (average = 3.29%). At least seven genes with known roles in limb development exhibit greatly divergent expression in the fore- and hind limbs of three of four species and among the limbs of all species (*Col2a1*, *Hoxa13*, *Mecom*, *Pitx1*, *Rarb*, *Tbx4*, *Wnt5a*, *Zbtb16*), and at least 5 more in two of four species (*Hoxd9*, *Hoxd13*, *Msx1*, *Prrx2*, *Shh*) (Additional file 17: Table S3). As described, we confirmed gene expression patterns of some of these divergently expressed genes (e.g., *Hoxa13* and *Hoxd13*) for some stages and species by *in situ* hybridization.

## Discussion

In this study we investigated the manner in which expression of the shared genes that regulate limb morphogenesis has been modified during the diversification of mammalian limb morphology. To do this we compared the patterns of gene expression in the fore- and hind limbs of developing bats, mice, opossums, and pigs using RNA-Seq analysis and WISH.

We first investigated the general pattern of divergence of the expression of shared genes among the fore- and hind limbs of a single species. While they vary among species, on the surface our heat-map results suggest that gene expression is generally more conserved at the earliest examined stage of limb development (i.e., ridge) than at later stages. Fore- and hind limb gene expression is most conserved at the ridge stage and diverges at the bud stage in mouse, at the paddle stage in opossum, and appears not to greatly diverge in pig at any examined stage of limb development (i.e., fore- and hind limb of a given developmental stage are more similar to each other than they are to their homologous limbs at other stages). However, while the ridge clusters are statistically significant in mouse, they are not significant in pig or opossum. The lack of significant clustering in opossum at the ridge or any other stage is not that surprising, given that comparable stages of opossum fore- and hind limb development occur at very different stages of overall development when dissimilar organism-wide processes are underway (e.g., skin formation). However, the lack of significant ridge clustering in pig, combined with the significant clustering at the pig bud and paddle stages, suggests that the ridge might not be the most conserved stage of pig development. The pattern in bat, in which the fore- and hind limbs of the paddle stage significantly cluster while those of the ridge and bud do not, also appears to deviate from the early conservation hypothesis. However, it is important to note that forelimb development is often slightly ahead (~12 h in mouse) of hind limb development in placental mammals such as mouse, pig, and bat. Therefore, using a single embryonic stage for the fore- and hind limbs of these species might have somewhat inflated their observed differences in within-species gene expression.

We next investigated the general pattern of divergence of the expression of shared genes among species. Heat-map results suggest that gene expression is conserved among species at the earliest stages of limb development (i.e., ridge), relative to later stages. Specifically, the clustering of species by gene expression during the fore- and hind limb ridge stage more closely follows their phylogenetic relatedness (i.e., bat + pig) [60] than at later stages. By the paddle stage of limb development, the clustering of species by gene expression more closely follows adult limb morphology, with the highly divergent bat fore- and hind limbs emerging as the outliers. However, while the expression patterns of all species were positively correlated, no specific clusters were significant. This suggests that while the general limb toolkit is conserved among species, the examined species vary in limb gene expression at all stages consistent with their divergent timing of limb development relative to overall development and disparate adult limb morphologies.

Taken together, heat-map results are consistent with gene expression patterns varying more among species during later than earlier stages of limb development, and therefore partially support our first working hypothesis. This part of our first hypothesis is further supported by our calculations of the among-species conservation of gene expression at each developmental stage. These results suggest that for both the fore- and hind limb, the earliest examined stage of limb development (i.e., ridge) displays a highly conserved pattern of gene expression across species that drops significantly as limb development proceeds. However, our heat-map results provide less support for the hypothesis that gene expression patterns vary more within species during later than earlier stages of limb development.

Results of our study of the transcriptomic age index (*TAI*_*s*_) vary across species and limbs. Of the 8 cases in which we examined transcriptomic age (2 for each species – 1 for the forelimb and 1 for the hind limb), 3 (37.5%; mouse forelimb, pig fore- and hind limb) fit an hourglass model of transcriptomic age, with the middle stage of limb development (i.e., bud) possessing the oldest genes and the youngest (i.e., paddle) and oldest (i.e., ridge) stages the youngest. Four cases (50%; opossum fore- and hind limb, bat hind limb, mouse hind limb) show the opposite pattern, with the middle stage of limb development (i.e., bud) having the youngest genes and the youngest (i.e., paddle) and oldest (i.e., ridge) stages the oldest, and 1 case (12.5%; bat forelimb) displays decreasing transcriptomic age throughout development. However, it is important to note that in every case (100%), transcriptomic age is younger at the latest (i.e., paddle) than earliest (i.e., ridge) stage of limb development.

Results of this study therefore do not support the existence of a phylotypic period of limb development that ends at the onset of chondrogenesis, but do support the hypothesis that gene expression patterns vary more among species during the later than earlier stages of limb development. As a result, these findings are consistent the hypothesis that the hierarchical nature of development translates into increasing variation among species as development progresses [33–39]. However, it is important to note that the temporal range of the proposed phylotypic period remains as controversial as the existence of the period itself [73]; some authors propose that the phylotypic period may extend throughout the period of organogenesis [41], while others argue that it may be restricted to the pharyngula stage (~E8.0 in mouse) [74], or to early somite segmentation (~E8.0 – 8.5 in mouse) [75], or to the tail-bud stage (~E9.5 – 10.5 in mouse) [76]. While morphological studies of limb development have tended to define the phylotypic period more broadly [43, 44], recent embryo-wide molecular studies in vertebrates suggest that the phylotypic period might actually be temporally restricted to the earliest stages of limb development (E8.0-E8.5) [51, 73]. At least the among species results of this study would be consistent with the existence of a phylotypic period for the limb that ends by onset of the bud stage, or roughly E10.5 in mouse.

Going beyond general patterns of gene expression, we also investigated the relative expression levels of specific genes in the fore- and hind limbs of single species and among the limbs of different species. Although we identified some limb-patterning genes (e.g., *Hand1*, *Pax1*, *Tbx4*, *Tbx5*) that are differentially expressed in the fore- and hind limbs of all examined species, these genes represent a small subset (0.5 to 8.4%) of the differentially expressed genes in all species. Some of these identified genes were well-supported in the literature. For example, a DGE-tag study of *Myotis ricketti* (Rickett’s big-footed bat) found *Tbx5* to be expressed more highly in the forelimb digits of fetal bats, while *Tbx4* was more highly expressed in the hindlimb digits [77]. *Tbx5* is well established to be forelimb restricted, while *Tbx4* is hindlimb restricted in mice, opossums, and chickens [23, 63, 64], providing support for our RNA-Seq results. We also identified a suite of key limb-patterning genes that are differentially expressed among the homologous limbs of all species. Therefore, results of this study are consistent with the hypothesis that the expression patterns of several limb-patterning genes significantly differ between the fore- and hind limbs of a single mammalian species and among the homologous limbs of different species.

The results of the RNA-Seq assays were generally supported by WISH from this study and previous studies. Results of this study confirmed that several *Hoxa* and *Hoxd* cluster genes exhibit distinct expression domains among species. Previous studies demonstrated that *Prrx1* (also known as *MHox* or *Prx1*) is expressed in similar domains during early stages of bat and mouse limb development, but that its’ expression expands in the hand-plate of bats relative to those of mice [11]. *Fgf8* is normally expressed in the AER (Apical Ectodermal Ridge) of mammalian limbs [78, 79]. Relative to mouse, *Fgf8* expression in pig has been shown to be reduced at later stages of limb development [8]. In contrast, bat forelimbs have previously been shown to have a wider *Fgf8* expression domain in the AER relative to the forelimbs of mice at similar stages [12].

Results also suggest that only a small subset of genes (1.79 to 6.87%) displays divergent expression within (e.g., between the fore- and hind limb of a single species) and among (e.g., between the forelimbs of two different species) species. This finding suggests that the genes that display divergent expression in the fore- and hind limb of a single species are not likely to also display divergent expression across the homologous limbs of multiple species. Therefore, in contrast to our working hypothesis, results of this study are consistent with a scenario in which evolutionary divergence of limb form within (i.e., of the fore- and hind limb) and among species (i.e., of the forelimbs) is driven by changes in the expression of different genes. Taken together, results of this study are consistent with a scenario in which a small subset of limb genes controls fore- vs. hind limb identity, and a small but different subset controls the evolution of limb morphology across multiple species. This result, especially when combined with the heat-map results described above, suggests that different processes might be controlling variation in limb gene expression within- and among- species.

While these results are intriguing, one potential caveat of this study is that we were forced to use heterogeneous methods to align the RNA-seq reads of our study species. We first attempted to align reads of all species directly to reference genomes. While this worked well (i.e., high alignment rate) for mouse, pig, and opossum, species for which high quality genomes are available, this did not work well for bat. As the bat species we used, *Carollia perspicillata*, does not have a published reference genome, we initially tried to align to the published reference genome for *Myotis lucifugus*, a phylogenetically-related bat species. However, only 7-9% of *Carollia* reads aligned with the *Myotis* genome (Additional file 14: Table S7). To overcome this, we generated a *de novo* transcriptome for *Carollia*, which increased the alignment rate to a much more reasonable ~80% for the bat reads, and used these data for subsequent analyses. While the use of different mapping techniques for bat versus pig, mouse, and opossum could potentially introduce bias into our analysis, previous studies suggest that the aligning methods we used under the conditions we used them do not result in noticeable differences [80]. Furthermore, if the difference in methods was introducing significant bias, then we would expect for bat reads to fall as outliers in all cross-species comparisons. Instead, we found that bat reads often clustered with pig or mouse to the exclusion of other species. Therefore, while it is important to note that we were forced to use heterogeneous methods to analyze our data, our data are not consistent with these different methods biasing our results. For further discussion, see Methods and Additional file 1.

## Conclusions

Using RNA-Seq, we generated transcriptomes for the ridge, bud, and paddle stages of limb development in mouse, opossum, bat, and pig to explore the hypothesis that mammalian limb diversification has occurred through the differential expression of shared conserved genes. Generally, there is greater variation among species at later stages (paddle) of development than at earlier stages (ridge). In addition, genes expressed at later stages tend to be younger in evolutionary age than those expressed at earlier (ridge) stages. Several key limb-patterning genes are differentially expressed among the homologous limbs of the species under study, though only a small subset of these shared genes are differentially expressed in the fore- and hind limbs of all species. In addition, only a small subset of these shared genes are differentially expressed in the fore- and hind limbs of the same species. Our results generally support the hypothesis that variation among species increases as development progresses, but do not support the presence of a phylotypic period of limb development that ends at chondrogenesis.

## Methods

*Embryo collection and staging –* All procedures were within the guidelines of the University of Illinois Institutional Animal Care and Use Committee (IACUC). Embryonic mice (ICR strain, Taconic) and opossums were obtained from timed matings [81, 82] in breeding colonies housed in the Sears Lab at the University of Illinois. Pig embryos were obtained through timed inseminations following ovulations at the University of Illinois pig farm [83]. For pig, mouse, and opossum, embryos were dissected out and placed in RNALater at 4 °C overnight before being frozen until the limbs were dissected off. Bat embryos were obtained from field collections in Trinidad and staged according to Cretekos et al. 2005 [57]. Forelimbs and hindlimbs were dissected and placed in RNALater (Qiagen) and stored at room temperature. Limbs from the ridge (Wanek stage 2), bud (Wanek stage 3/4), and paddle (Wanek stage 6) stages of development were obtained for all species [56]. In bat these stages fall around *Carollia* stages (CS) 13, 14, and 15 for both the fore- and hind limb, in mouse around embryonic days (E) 10, 10.5, and 11.5 for both the fore- and hind limb, in opossum around stages 27, 28, and 29 for the forelimb, and 30, 31, and 32 for the hind limb, and in pig around embryonic days (E) 21.5, 23.5, and 25.5 for both the fore- and hind limb [57, 84–87]. At least three biological replicates were collected for each species/stage, though some were excluded from analysis post-library construction. Forelimbs from a single embryo and hindlimbs from a single embryo were combined for the bud and paddle stages. For ridge stages, limbs from 2 embryos were pooled.

Embryos for whole-mount *in situ* hybridization (WISH) were collected at the appropriate stages, fixed in 4% paraformaldehyde (PFA) overnight and dehydrated through a methanol series before being processed for WISH (see below).

*RNA sequencing* - Limbs were removed from embryos as above and stored in RNALater (Life Technologies) at −20 °C until further processing. RNA was extracted from tissues using the E.Z.N.A. Total RNA Kit I (OMEGA bio-tek #R6834), and converted into RNASeq libraries with the Illumina TruSeq RNA Sample Preparation Kit (Illumina RS-122-2001). Before RNASeq library construction, a few RNA samples were checked for quality (Bioanalyzer). Libraries were sequenced on an Illumina HiSeq 2500 housed in the Roy G. Carver Biotechnology Center at the University of Illinois under the supervision of Dr. Alvaro Hernandez. For all species, the resulting single-end RNASeq sequences were pre-processed to remove Illumina adaptors and low quality bases (score <20) from the 3’ read end. The pre-processed libraries where then aligned to their corresponding reference genomes. For mouse, opossum, and pig, we used the Ensembl reference genomes and annotation files corresponding to assemblies, GRCm38, BROADO5, and Sscrofa10.2, respectively. As the bat species we are using (*Carollia perspicillata*) does not have a published genome, we initially used *Myotis lucifugus* (The Little Brown Bat, whose genome was sequenced [88]) as a reference genome (Myoluc2.0), but the alignment rate with TopHat [89] for *Carollia* sequences was very low (~7-9% for bud and paddle stages). Therefore, we next performed a *de novo* transcriptome assembly with *Carollia* reads. To do this, we pooled all bat libraries and fed them into Trinity [90], a *de-novo* assembly tool. Trinity has been used by other groups to assemble bat RNASeq transcriptomes from various tissues [91], and several bat mitochondrial genomes ([92]. Trinity has become increasingly popular for other species for which a genome is not available [80]. Pooled cleaned libraries were combined into a single file, and duplicated sequences removed to reduce computational requirements. Trinity predicted 350,733 gene transcripts which were filtered to keep only those matching the protein sequences of the SwissProt database (blastx, E-value < 1e-20) [93]. This resulted in 88,930 gene isoforms. These were used as a reference to map the RNA-Seq libraries. For bat, read alignment and gene expression were computed with RSEM [94] using as reference sequences the 88,930 gene isoforms previously described. RNA-Seq data has been deposited in the NCBI Gene Expression Omnibus (GEO) under accession number GSE71390 [53].

For species with a reference genome (mouse, opossum, and pig) we aligned the reads using STAR (Spliced Transcripts Alignment to a Reference) [95] and then used Cufflinks [89] to compute their gene expression. For all four species, gene expression values were normalized as fragments per kilobase of transcript per million mapped reads (FPKM). To reduce noise when quantifying FPKM values we used all reads aligned to genes, and did not discard reads aligned to non-constitutive exons or non-orthologous gene fragments. To account for differences in the mass composition between samples we used DESeq normalization as described by [96]. All analyses were performed in R [97].

The aligner used for mouse, pig, and opossum (STAR), could not be used for the bat species *Carollia* because a species-specific reference genome for *Carollia* is not available. Instead, BOWTIE, which is internally used by RSEM, was used. A comparative analysis of various aligners, including STAR and TOPHAT (which, like RSEM, relies on BOWTIE for alignment), shows that noticeable differences between aligners only result when reads are mapped to unannotated junctions [80]. In our methods we did not use unannotated junctions (bat reads were aligned against the *de novo* transcriptome, and all other species were aligned only to annotated reference genes). Thus, using BOWTIE for bat and STAR for all other species does not introduce a significant source of bias in our analyses.

*Analyses, Divergence of the expression pattern of shared genes within and among species* - To identify when the expression pattern of shared genes diverges among the fore- and hind limbs of a single species and among the limbs of different species, we first graphically visualized the similarity in expression of a set of 6,583 genes (orthologous to all four species) in all four species (bat, mouse, opossum, pig) at each stage of limb development (ridge, bud, paddle) using a series of heat-map analyses, where distances between any to gene expression profiles were computed using Spearman correlation coefficients. Testing for the significance of the clustering was done using pvclust [59]. To determine these orthologous genes, the transcriptome from *Carollia* was used as a reference to align the transcriptomes of mouse, pig, and opossum (blastn, E-value 1e-20). Bat genes matching more than one orthologous gene in any other species were filtered out, and then the bat genes that have orthologous sequences in all the other species determined. This gave a set of 6,583 genes found in all 4 species.

We then used the set of 6,583 genes (orthologous to all four species) to calculate the among-species conservation of gene expression at each developmental stage, using the mean of all species pairwise Spearman coefficients (c):

$$ c=\frac{1}{\left({}_2^k\right)}{\displaystyle \sum_{i=1}^{k-1}}{\displaystyle \sum_{j> i}^k}\kern0.5em {r}_{i, j} $$

Where r _i, j_ is the Spearman coefficient between species i and j at a given stage, and

k is the total number of species under study. We used the Spearman rather than Pearson coefficient, as the former is more robust against outliers. To test the robustness of any differences in Spearman coefficients among species with respect to the selection of orthologous genes, we randomly sub-sampled 500 sets of genes at early and late stages at intensities ranging from 50 to 100% of all genes.

To investigate the divergence of the expression pattern of shared genes among species, we computed the evolutionary age of the genes of each species. To do this we determined the most distant phylogenetic node among bat, pig, mouse, and opossum that contained a detectable homologue for each gene. FASTA sequences of all species genes were pooled to form a single database to which each species genome was aligned (blast hit, E-value 1e-5 for homologue detection) [47]. In this analysis, youth corresponds to genes that have more recently evolved and, therefore, are more likely to be specific to a single species. Conversely, old genes are those that have been inherited from a common ancestor and are present among all descendant species. The specificity of the transcriptome of each species was then quantified across limb development (ridge, bud, paddle) using the transcriptome age index (*TAI*), which is the sum of each gene evolutionary age weighted by its expression [49]. As a result, a smaller *TAI* value corresponds to a younger age, and a larger *TAI* value an older age. For a given species at a given stage, *s*, *TAI*_*s*_ is mathematically defined as:

$$ T A{I}_s=\frac{{\displaystyle {\sum}_{i=1}^n}\; p{s}_i{e}_i}{{\displaystyle {\sum}_{i=1}^n}\kern0.5em  ei} $$

Here, *ps*_*i*_ and *e*_*i*_ are the age and expression values of gene *i*, respectively. The total number of genes is represented by *n*. We then analyzed the statistical significant of the *TAIs* trend of each species using the procedure proposed by [47], in which the variance of *TAIs* across stages (ridge, budge, and paddle), *VTAI*, is used as a test statistic. The null distribution for this analysis was obtained by sampling 1000 *VTAI* surrogates. Each surrogate was generated by randomly permuting the *ps* assignations. The null distribution was modeled as a gamma distribution, and its parameters estimated using the MASS library in R [98].

*Similarity of gene expression patterns within species* – To determine the degree to which patterns of fore- and hind limb gene expression divergence are similar between species we identified those genes with the most divergent expression (75% percentile and above) between the fore- and hind limb for each species. We then performed a series of pairwise comparisons in which we compared the resulting lists of genes for two species and determined the percentage of the total number of genes that are present in both.

*Similarity of gene expression patterns within and among species* – We also determined the degree to which patterns of gene expression divergence are similar within (e.g., between the fore- and hind limb of a single species) and among (e.g., between the forelimbs of two different species) species. To do this we identified those genes with the most divergent expression (75% percentile and above): between the fore- and hind limb for each species (within species, as described above), among the forelimbs of all species (among species), and among the hind limbs of all species (among species). We then compared the within and among species lists and determined the percentage of the total number of genes that are present in both. We also used the DAVID Ontology database (http://david.abcc.ncifcrf.gov/) to identify a subset of genes from the lists with known roles in limb development [99, 100].

### Whole-mount *in situ* hybridization (WISH)

To confirm the RNA-Seq expression data of some of our divergently expressed candidate genes, WISH was performed on embryos at the ridge, bud and paddle stages for the species investigated (See Additional file 15: Table S6). Often, mouse differs enough from bat and opossum that it is necessary to make species-specific probes for WISH. Coding sequences were found in the NCBI Nucleotide database and primers designed specific to each species using NCBI PrimerBLAST [101]. (See Additional file 16: Table S4 for accession numbers and primer sequences). The primers were synthesized by Sigma-Aldrich. RNA was purified from bat and opossum limbs and used to generate cDNA using the SuperScript III Reverse Transcriptase kit (Invitrogen) following the manufacturer’s instructions. PCR was done to amplify the cDNA for the gene of interest using the species-specific primers and standard PCR methods. Genes were cloned into pGem T easy (see Additional file 16: Table S4 for primer sequences and accession numbers) and sequenced. After sequencing the direction and sequence identity were confirmed using NCBI Blast. Next, plasmid DNA was linearized with the appropriate restriction enzyme (*NotI* or *SpeI*) and an in vitro transcription reaction to generate antisense mRNA probes was performed using Roche or Promega reagents. Probes were all digoxigenin labeled. After synthesis, probes were purified by ethanol precipitation and checked on a NanoDrop for concentration and RNA Quality. Probes were stored at −80 °C until use.

To perform the *in situ* hybridization, standard methods were based on the following protocols [102–105]. BM Purple (Roche) was used to develop the reactions. After development (assessed by purple/blue staining where the probe has bound), the embryos were photographed on a Leica camera microscope and fixed in 4% PFA for long-term storage.

## Additional files

Additional file 1: Library quality control and note on bat aligment. (DOCX 29 kb)

Additional file 2: Figure S1. Gene Expression Distributions Within & Between Species. Box and violin plots of FPKM (Fragments per kilobase per million reads) for each sample. 1e-3 FPKM was defined as the minimum detectable expression value. Violin plots (gray areas) of the density of reads have a bimodal distribution, one corresponding to genes with zero expression (below the cutoff) and the other to active genes. Boxplots indicate that all libraries have similar distribution of gene expression. The x-axis shows each individual sample used in the analysis. A: Forelimb and hindlimb of bats. B: Forelimb and hindlimb of mouse. C: Forelimb and hindlimb of pig. D: Forelimb and hindlimb of opossum. (TIF 7475 kb)

Additional file 3: Figure S2. Analysis of consistency among replicates. Hierarchical clustering was used to determine similarity between replicates. A: All samples for bat. B: All samples for mouse. C: All samples for pig. D: All samples for opossum. (TIF 8736 kb)

Additional file 4: Figure S3. Statistical significance of hierarchical clustering of all stages and limbs of each species (Fig. 2 in text). Red boxes are statistically significant clusters. A: Bat, B: Mouse, C: Opossum, D: Pig. The R package pvclust was used to determine statistical significance. FL = forelimb, HL = hindlimb. In A, St13, St14, and St15 correspond to the ridge, bud, and paddle stages respectively. In B, StW2, StW3-4, and StW6 correspond to the ridge, bud, and paddle stages. In C, St27 and St30, St28 and St31, and St29 and St32 correspond to the ridge, bud and paddle. For D, St20, St22, and St26 correspond to the ridge, bud and paddle stages. (TIF 1185 kb)

Additional file 5: Figure S4. Statistical significance of hierarchical clustering of pairwise Spearmann coefficient values on fore (A-C) and hind (D-F) limbs (Fig. 3 in text). The pvclust R package was used to test for significance. No clusters were significant (*p*-value <=0.05). Abbreviations for stages are as in Supplementary Figure 3. (TIF 1270 kb)

Additional file 6: Figure S5. Variation of transcriptome age index (VTAI) surrogate distribution for forelimbs. Bat, mouse, and pig forelimb trends are highly significant, while opossums are not. A: Bat B: Mouse C: Pig D: Opossum. (TIF 2530 kb)

Additional file 7: Figure S6. Variation of transcriptome age index (VTAI) surrogate distribution for hindlimbs. As with the hindlimbs, bat, mouse, and pig forelimb trends are highly significant, while opossums are not. A: Bat B: Mouse C: Pig D: Opossum. (TIF 2474 kb)

Additional file 8: Table S1. Genes Divergently Expressed in Forelimbs vs. Hindlimbs. (DOCX 28 kb)

Additional file 9: Table S2. Genes with known roles in limb development (as identified by DAVID) that exhibit greatly divergent expression (75th percentile and above) among the limbs of all species. (DOCX 16 kb)

Additional file 10: Figure S10. *Hoxd12* WISH for opossum and bat forelimb and hindlimb. (TIF 23437 kb)

Additional file 11: Figure S7. Additional replicates of *Hoxd13 *WISH for mouse, bat, and opossum forelimb and hindlimb. (TIF 28408 kb)

Additional file 12: Figure S8. Additional replicates of *Evx2* WISH for mouse and opossum forelimb and hindlimb. In mouse hindlimb ridges, ventral side is shown to highlight that purple staining representing *Evx2* expression is not in the limb. (TIF 30642 kb)

Additional file 13: Figure S9. Additional replicates of *Hoxa13* WISH for mouse and opossum forelimb and hindlimb. (TIF 29860 kb)

Additional file 14: Table S7. Alignment of bat reads to the *Myotis lucifugus* genome using TOPHAT. FL and HL correspond to forelimb and hindlimb. (DOCX 29 kb)

Additional file 15: Table S6. Embryo samples used for whole-mount in situ hybridization. (DOCX 15 kb)

Additional file 16: Table S4. Accession Numbers & Primer Sequences For WISH. (DOCX 15 kb)

Additional file 17: Table S3. Genes with known roles in limb development that exhibit greatly divergent expression in the fore- and hind limbs of a single species and among the limbs of all species. (DOCX 20 kb)

Additional file 18: Table S5. Samples Used in Final Analysis. (DOCX 21 kb)

## Abbreviations

AER: Apical ectodermal ridge
CS: Carnegie stage
DAVID: Database for annotation, visualization, and integrated discovery
DGE-tag: Digital gene expression tag
FL: Fore limb
FPKM: Fragments per Kilobase of transcript per million mapped reads
HL: Hind limb.
MASS: Modern applied statistics with S
NCBI: National center for biotechnology information
PCR: Polymerase chain reaction
PFA: Paraformaldehyde
ps: Phylostratum
RNA-Seq: RNA sequencing
RSEM: RNA-Seq by Expectation Maximization
STAR: Spliced transcripts alignment to a reference
TAI: Transcriptome age index
VTAI: Variance of TAIs
WISH: Whole mount *in situ* hybridization
