# Supplementary material for: Transcriptomic insights into the genetic basis of mammalian limb diversity
Source: BMC Evol Biol. 2017 Mar 23;17:86. doi: 10.1186/s12862-017-0902-6 (PMC5364624; doi:10.1186/s12862-017-0902-6)
Supplement: Supplementary file 1 — Library quality control and note on bat aligment. (DOCX 29 kb) [file 12862_2017_902_MOESM1_ESM.docx]

**Additional file**:

*Quality Control of RNA-Seq Data-Library Size, Alignment and Gene Expression*: After *de novo* transcriptome assembly with Trinity [1] of the *Carollia* sequences (resulting in 88,930 transcript sequences), the RNA-Seq reads from all stages were aligned using RSEM [2]. This increased the alignment from ~9% (attempting to align reads to *Myotis lucifugus* genomic sequences [3]) by an order of magnitude, to ~80%. The average library size for bat was 35M. For a summary of all species alignment and read data, see Supplementary Table 5. Mouse library size was 34M and had an alignment rate of ~98% with the mouse genome. Opossum library size was 43M and with ~91% alignment, and pig library size was 38M with ~92% alignment. While we aimed to have 3 biological replicates per species per stage, some samples were discarded after initial library preparation due to low quality. For bat samples, it was not practical to replace them with a fresh one as they must be collected in the field, therefore we had only 2 of each FL and HL for the paddle stage (Stage 15). For mouse, one HL ridge stage and one FL bud stage were discarded, while we had 4 HL paddle stages in the analysis. For the opossum no samples were discarded but we had 6 FL buds and 5 HL buds. Pig samples had only 1 FL ridge and 2 HL ridges, while we had 4 each of FL and HL paddle stages. Due to the limited availability of pig embryos, it was not possible to replace bad samples.

FPKM was used to calculate expression, with RSEM for bats and Cufflinks for other species. We examined gene expression distributions within and between species. 1e-3 FPKM was defined as a minimum detectable expression value, thus expression values below this cutoff were considered to be unexpressed. FPKM values were log-transformed to make them comparable and plotted on box/violin plots (Supplementary Figure 1). The libraries were distributed within the same range (-7 to 15log(FPKM) and have a bimodal distribution which corresponds to inactive (not detectable) and active genes, regardless of species and developmental stage. Thus, the different software packages used to call gene expression did not produce noticeable differences in terms of distribution range.

Hierarchical clustering was used to determine the similarity between replicates, giving us a readout of consistency between biological replicates. Expression values were assigned a minimum detectable threshold, as described above, log-transformed, and then expression values for each gene were scaled across replicates to have mean zero and standard deviation. In general, there were 3 main clusters for each species that corresponded to early, middle, and late developmental stages (Supplementary Figure 2). In mouse and pig (Supplementary Figure 2B,C) early and late development, rather than FL or HL, formed mostly exclusive clusters, while middle stages were with either early or late. In opossum (Supplementary Figure 2D), the FL and HL formed a single cluster at early stages, but different clusters at later stages. For bats (Supplementary Figure 2A) FL and HL clustered separately at early stages but together at later ones. It appears that between replicates, results are consistent based on clustering. This RNA-Seq data was deposited in GEO under accession number GSE71390 and was referenced in [4].

*A note on bat alignment using Trinity*: In our current mapping method, bat libraries were mapped against its *de novo* transcriptome (generated in Trinity) using BOWTIE (used internally by RSEM) whereas mouse, pig, and opossum libraries were mapped against their reference genomes using STAR, a splice- tolerant aligner. We did this as there is no reference genome available for this species of bat (*Carollia perspicillata*), making inappropriate the use of STAR. The use of two aligners may affect the biological interpretations when comparing the results of bat and the other species. This is an important point and, therefore, the use of alternative mapping strategies must be assessed.

This alternative mapping strategy requires the use of a reference genome for each species. For bat, we used the reference genome of *Myotis lucifugus*, a phylogenetically close species to *Carollia perspicillata*. We used TOPHAT, a splice-tolerant aligner, which --like RSEM-- it is based on BOWTIE. Unfortunately, after mapping bat’s fore- and hindlimb libraries of the bud and paddle stages, we found that the alignment rates were very low (7-9%; see Supplementary Table 7), making this strategy unusable to detect the expression of bat genes. Therefore we assembled the transcriptome in Trinity and used RSEM to align bat reads. Given these results, we concluded that our current mapping strategy assuages the introduction of biases in our interpretations compared to the present-day alternative of using reference genomes for all species.

**Figure Legends**:

**Figure S1: Gene Expression Distributions Within & Between Species**. Box and violin plots of FPKM (Fragments per kilobase per million reads) for each sample. 1e-3 FPKM was defined as the minimum detectable expression value. Violin plots (gray areas) of the density of reads have a bimodal distribution, one corresponding to genes with zero expression (below the cutoff) and the other to active genes. Boxplots indicate that all libraries have similar distribution of gene expression. The x-axis shows each individual sample used in the analysis. A: Forelimb and hindlimb of bats. B: Forelimb and hindlimb of mouse. C: Forelimb and hindlimb of pig. D: Forelimb and hindlimb of opossum.

**Figure S2: Analysis of consistency among replicates**. Hierarchical clustering was used to determine similarity between replicates. A: All samples for bat. B: All samples for mouse. C: All samples for pig. D: All samples for opossum.

**Figure S3:** Statistical significance of hierarchical clustering of all stages and limbs of each species (Figure 2 in text). Red boxes are statistically significant clusters. A: Bat, B: Mouse, C: Opossum, D: Pig. The R package pvclust was used to determine statistical significance. FL = forelimb, HL = hindlimb. In A, St13, St14, and St15 correspond to the ridge, bud, and paddle stages respectively. In B, StW2, StW3-4, and StW6 correspond to the ridge, bud, and paddle stages. In C, St27 and St30, St28 and St31, and St29 and St32 correspond to the ridge, bud and paddle. For D, St20, St22, and St26 correspond to the ridge, bud and paddle stages.

**Figure S4**: Statistical significance of hierarchical clustering of pairwise Spearmann coefficient values on fore (A-C) and hind (D-F) limbs (Figure 3 in text). The pvclust R package was used to test for significance. No clusters were significant (p-value <=0.05). Abbreviations for stages are as in Supplementary Figure 3.

**Figure S5: Variation of transcriptome age index (VTAI) surrogate distribution for forelimbs**. Bat, mouse, and pig forelimb trends are highly significant, while opossums are not. A: Bat B: Mouse C: Pig D: Opossum

**Figure S6: Variation of transcriptome age index (VTAI) surrogate distribution for hindlimbs**. As with the hindlimbs, bat, mouse, and pig forelimb trends are highly significant, while opossums are not. A: Bat B: Mouse C: Pig D: Opossum

**Figure S7**: Additional replicates of *Hoxd13* WISH for mouse, bat, and opossum forelimb and hindlimb.

**Figure S8**: Additional replicates of *Evx2* WISH for mouse and opossum forelimb and hindlimb. In mouse hindlimb ridges, ventral side is shown to highlight that purple staining representing *Evx2* expression is not in the limb.

**Figure S9**: Additional replicates of *Hoxa13* WISH for mouse and opossum forelimb and hindlimb.

**Figure S10**: *Hoxd12* WISH for opossum and bat forelimb and hindlimb.

**References**:

1. Grabherr MG, Haas BJ, Yassour M, Levin JZ, Thompson DA, Amit I, Adiconis X, Fan L, Raychowdhury R, Zeng Q *et al*: **Full-length transcriptome assembly from RNA-Seq data without a reference genome**. *Nature biotechnology* 2011, **29**(7):644-652.

2. Li B, Dewey CN: **RSEM: accurate transcript quantification from RNA-Seq data with or without a reference genome**. *BMC bioinformatics* 2011, **12**:323.

3. Lindblad-Toh K, Garber M, Zuk O, Lin MF, Parker BJ, Washietl S, Kheradpour P, Ernst J, Jordan G, Mauceli E *et al*: **A high-resolution map of human evolutionary constraint using 29 mammals**. *Nature* 2011, **478**(7370):476-482.

4. Sears KE, Maier JA, Rivas-Astroza M, Poe R, Zhong S, Kosog K, Marcot JD, Behringer RR, Cretekos CJ, Rasweiler JJt *et al*: **The Relationship between Gene Network Structure and Expression Variation among Individuals and Species**. *PLoS genetics* 2015, **11**(8):e1005398.
