# Supplementary figures and images for: Transcriptomic insights into the genetic basis of mammalian limb diversity
Source: BMC Evol Biol. 2017 Mar 23;17:86. doi: 10.1186/s12862-017-0902-6 (PMC5364624; doi:10.1186/s12862-017-0902-6)

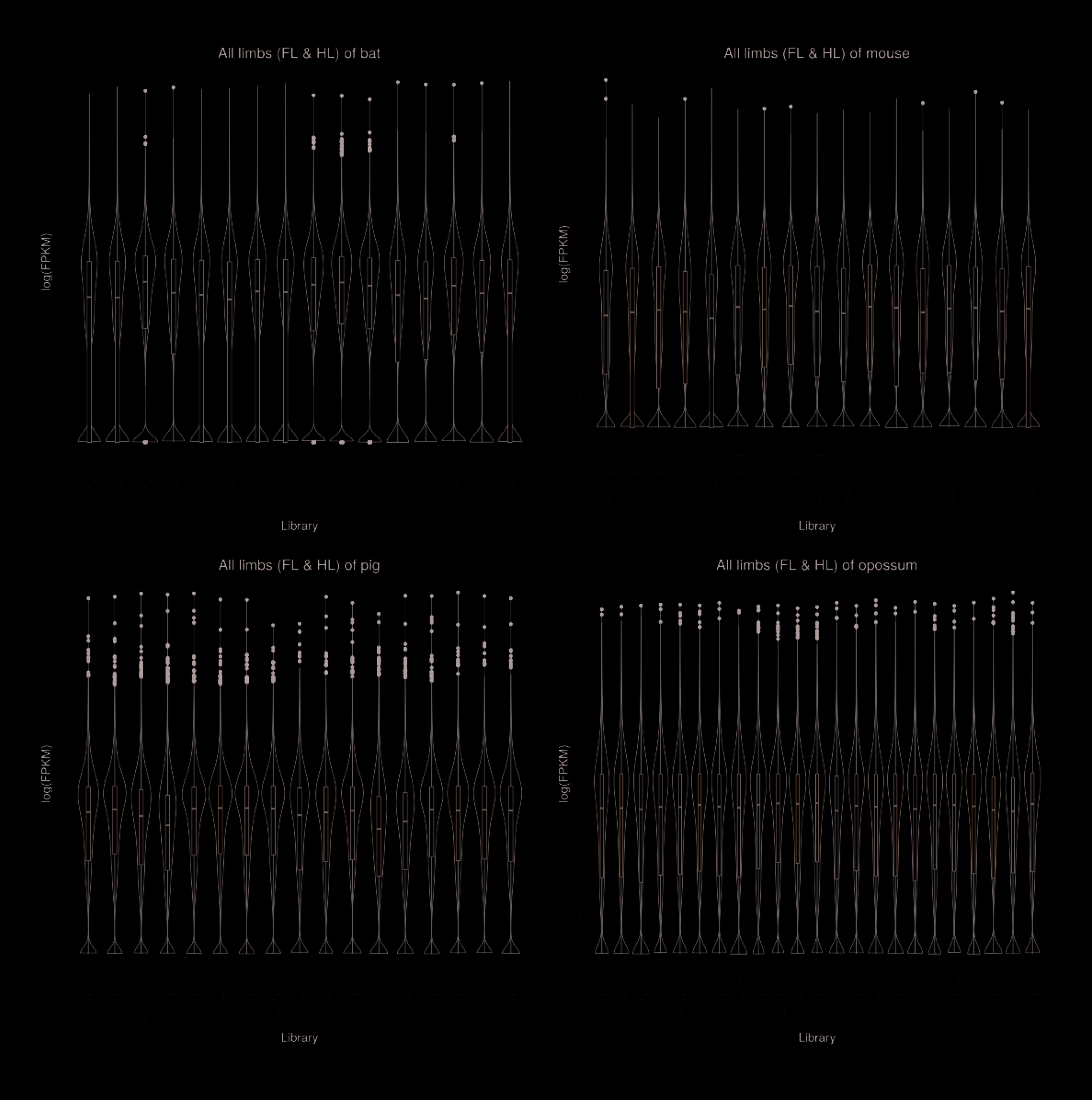

Supplement: Supplementary file 2 — Gene Expression Distributions Within & Between Species. Box and violin plots of FPKM (Fragments per kilobase per million reads) for each sample. 1e-3 FPKM was defined as the minimum detectable expression value. Violin plots (gray areas) of the density of reads have a bimodal distribution, one corresponding to genes with zero expression (below the cutoff) and the other to active genes. Boxplots indicate that all libraries have similar distribution of gene expression. The x-axis shows each individual sample used in the analysis. A: Forelimb and hindlimb of bats. B: Forelimb and hindlimb of mouse. C: Forelimb and hindlimb of pig. D: Forelimb and hindlimb of opossum. (TIF 7475 kb) [file 12862_2017_902_MOESM2_ESM.tif]

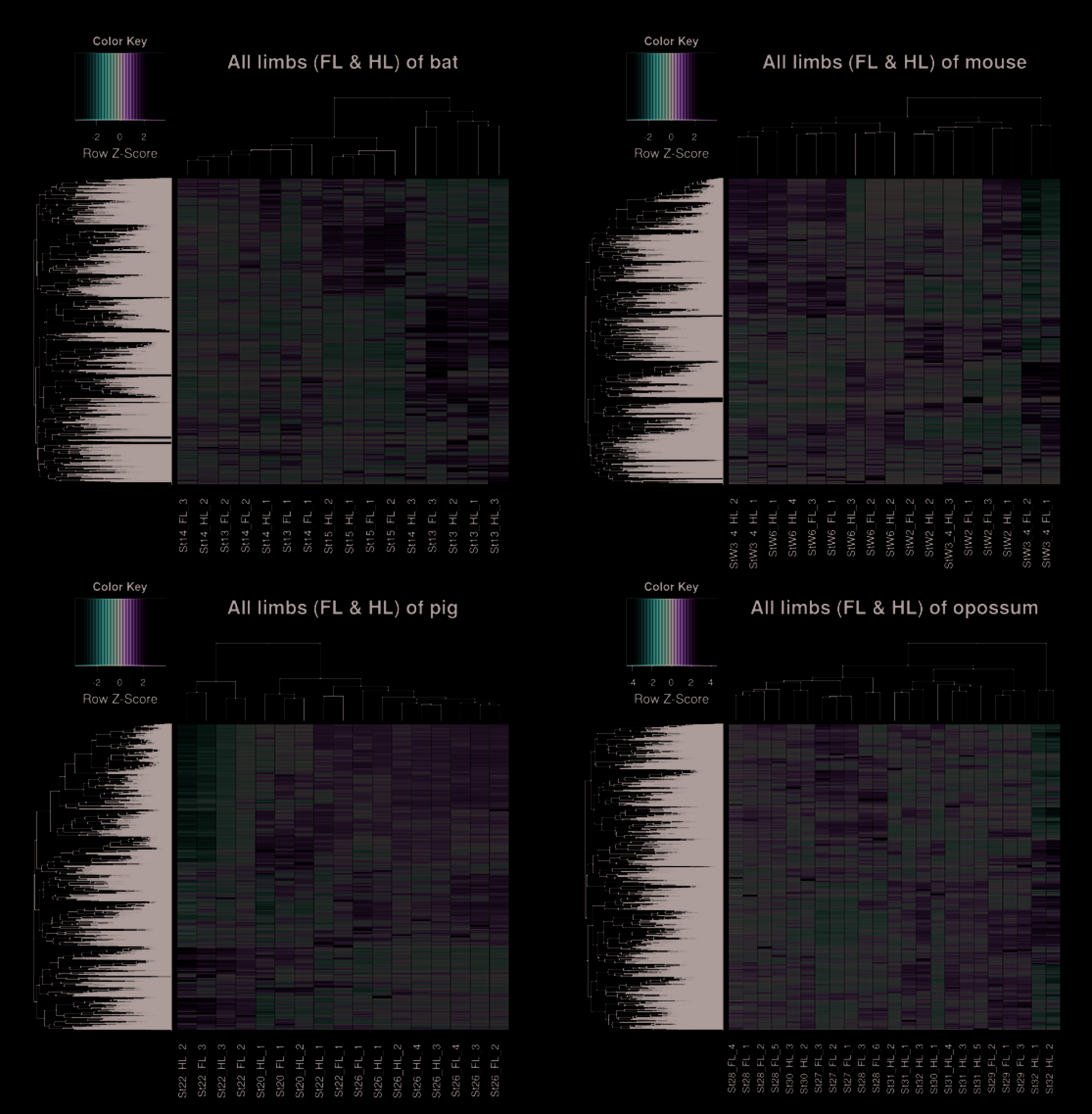

Supplement: Supplementary file 3 — Analysis of consistency among replicates. Hierarchical clustering was used to determine similarity between replicates. A: All samples for bat. B: All samples for mouse. C: All samples for pig. D: All samples for opossum. (TIF 8736 kb) [file 12862_2017_902_MOESM3_ESM.tif]

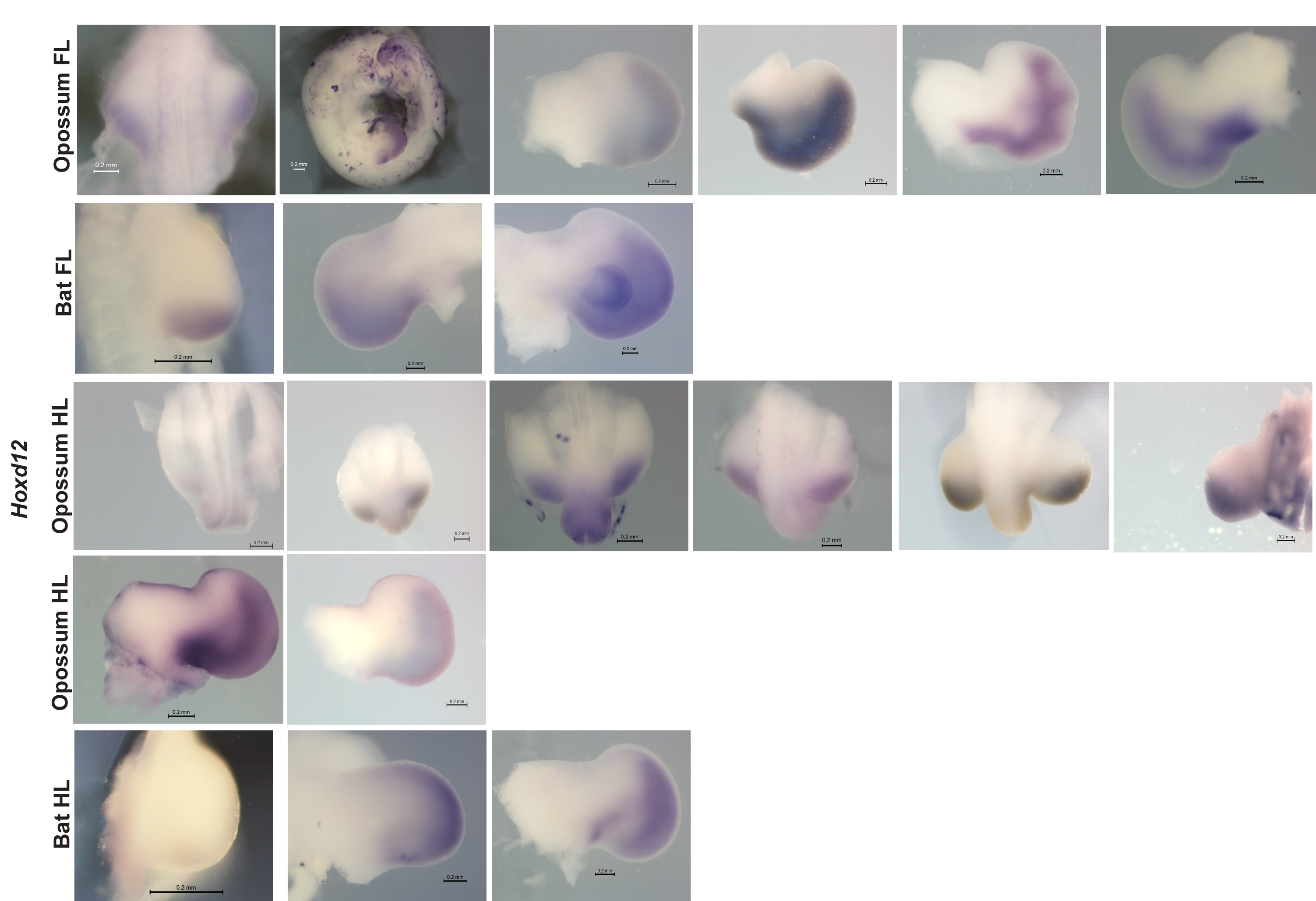

Supplement: Supplementary file 10 — Hoxd12 WISH for opossum and bat forelimb and hindlimb. (TIF 23437 kb) [file 12862_2017_902_MOESM10_ESM.tif]

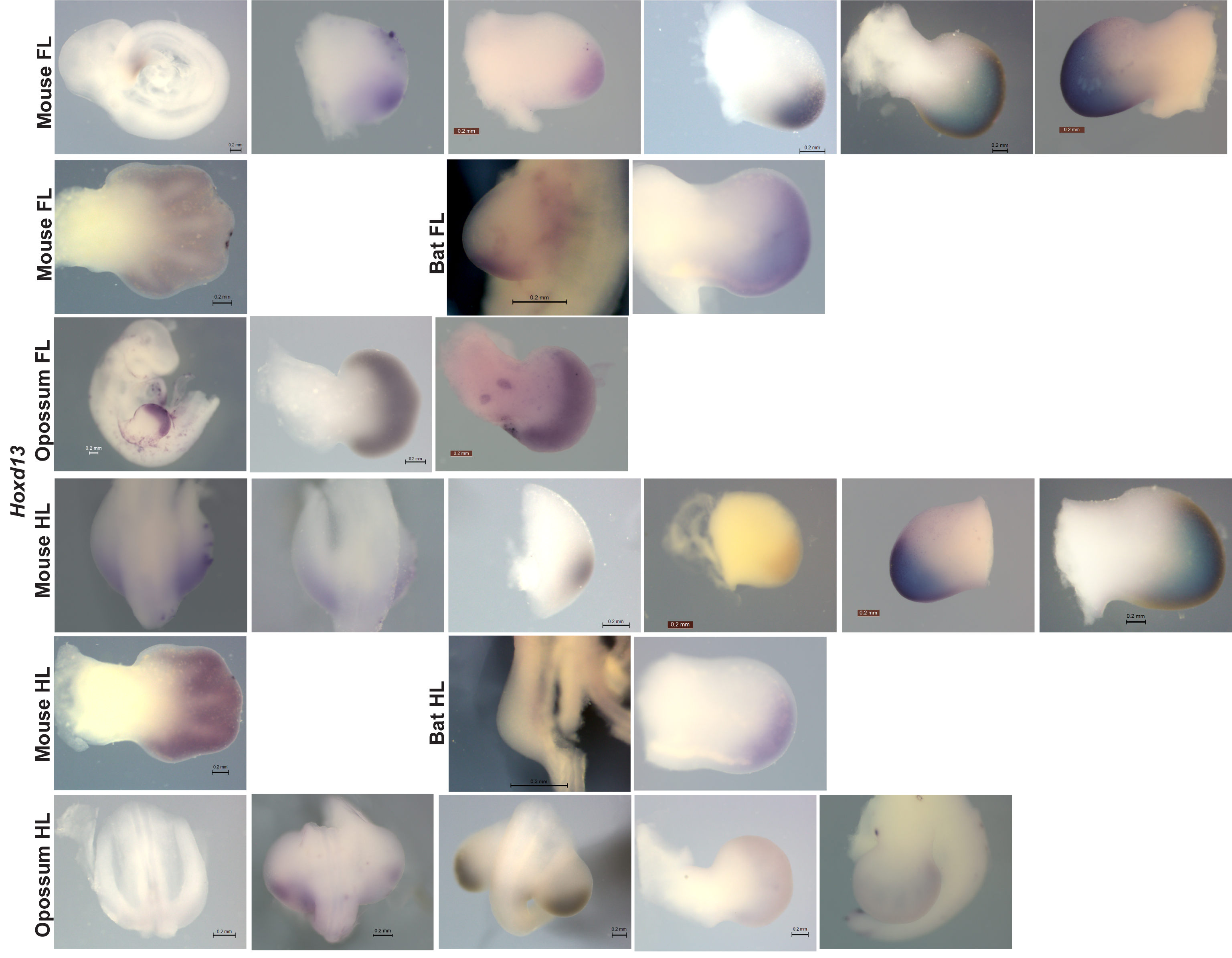

Supplement: Supplementary file 11 — Additional replicates of Hoxd13 WISH for mouse, bat, and opossum forelimb and hindlimb. (TIF 28408 kb) [file 12862_2017_902_MOESM11_ESM.tif]

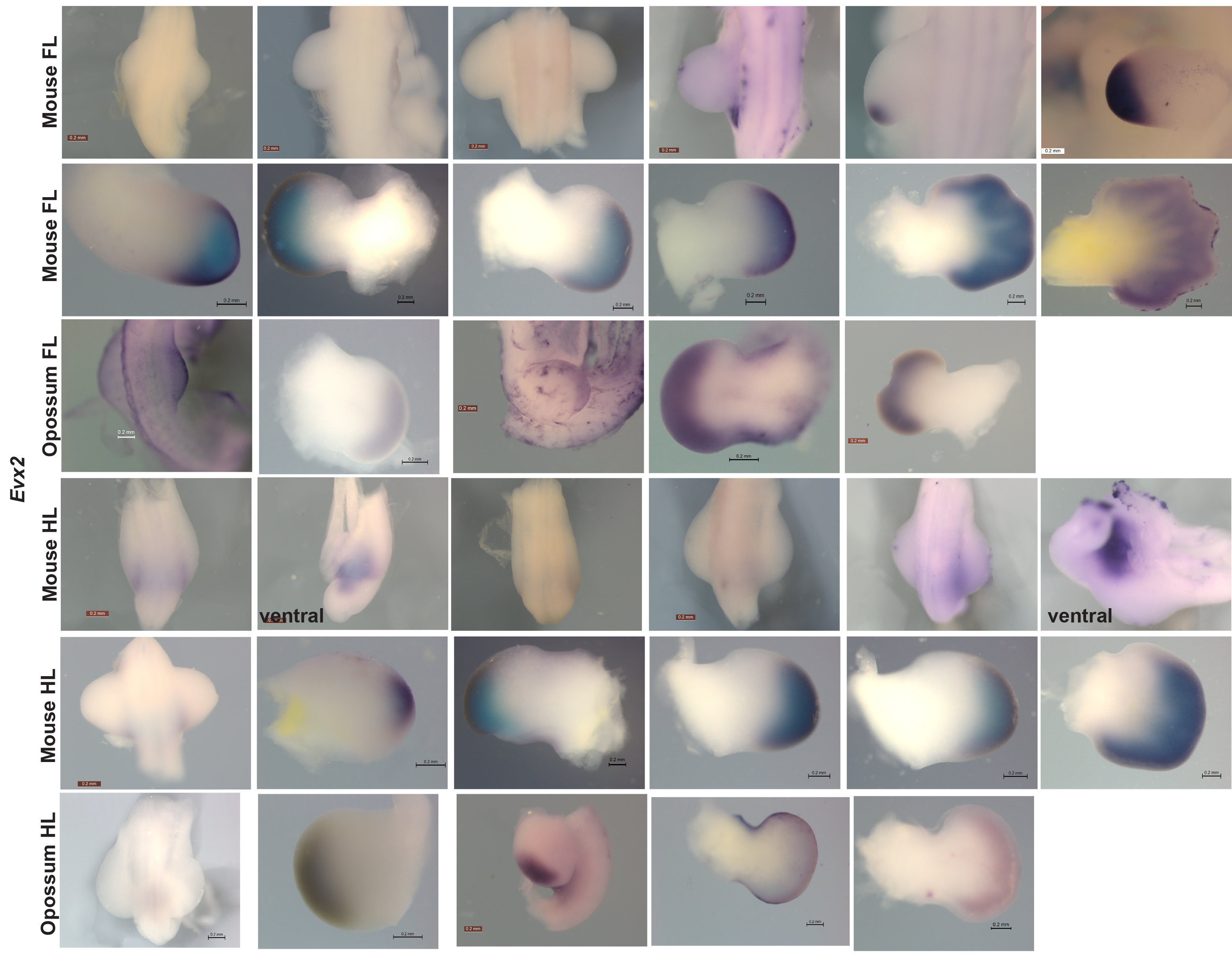

Supplement: Supplementary file 12 — Additional replicates of Evx2 WISH for mouse and opossum forelimb and hindlimb. In mouse hindlimb ridges, ventral side is shown to highlight that purple staining representing Evx2 expression is not in the limb. (TIF 30642 kb) [file 12862_2017_902_MOESM12_ESM.tif]

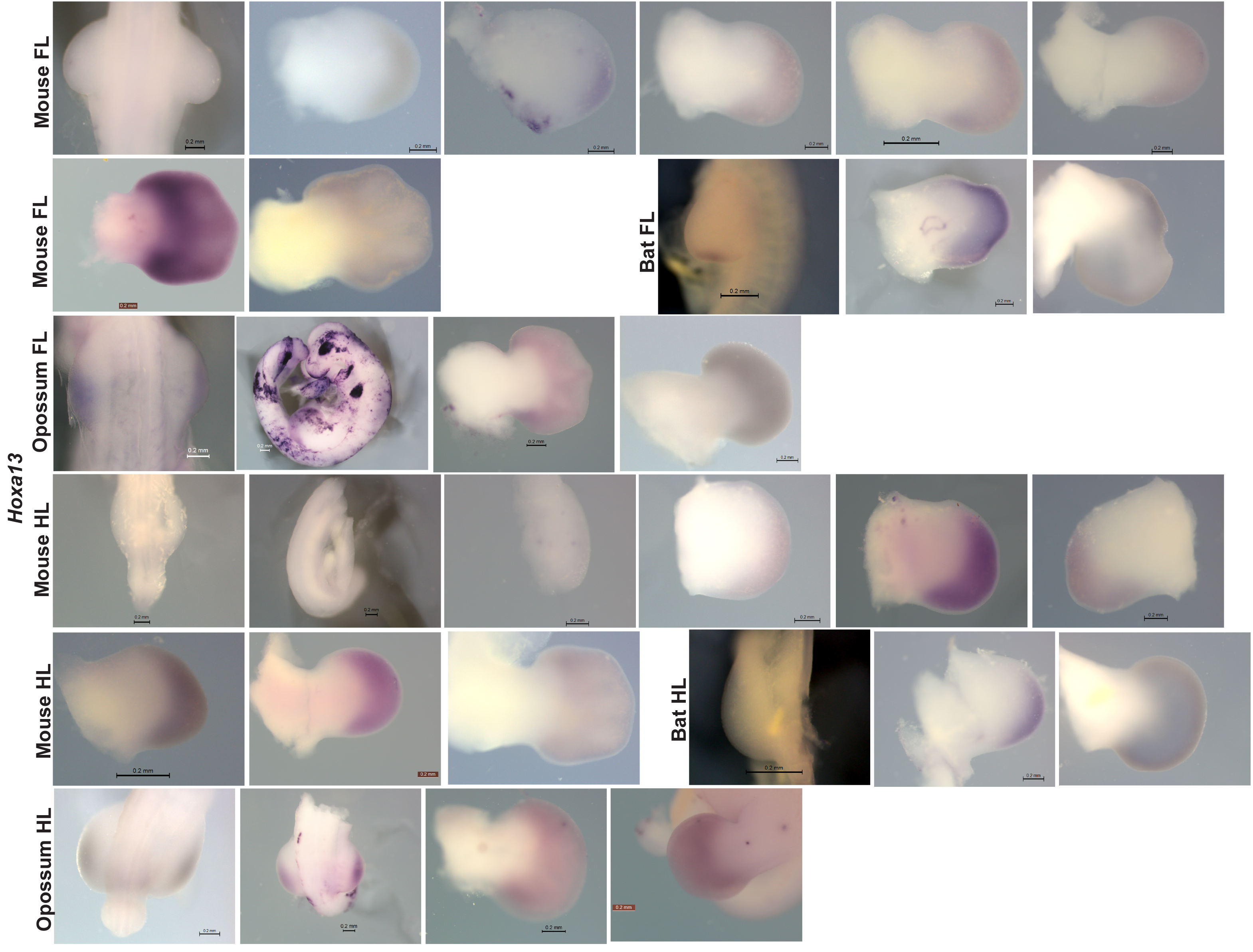

Supplement: Supplementary file 13 — Additional replicates of Hoxa13 WISH for mouse and opossum forelimb and hindlimb. (TIF 29860 kb) [file 12862_2017_902_MOESM13_ESM.tif]
