# Supplementary material for: Transcriptomic insights into the genetic basis of mammalian limb diversity
Source: BMC Evol Biol. 2017 Mar 23;17:86. doi: 10.1186/s12862-017-0902-6 (PMC5364624; doi:10.1186/s12862-017-0902-6)
Supplement: Supplementary file 8 — Genes Divergently Expressed in Forelimbs vs. Hindlimbs. (DOCX 28 kb) [file 12862_2017_902_MOESM8_ESM.docx]

Table S1: Genes Divergently Expressed in Forelimbs vs. Hindlimbs

| RIDGE | BUD | PADDLE | ALL STAGES |
| --- | --- | --- | --- |
| *A1cf* | *Apoe* | *Actn2* | *Hand1* |
| *Actn2* | *C1qc* | *Alpk2* | *Isl1* |
| *Apoa1* | *Ccbe1* | *Alx1* | *Myog* |
| *Asb9* | *Cntn4* | *Aspn* | *Pax1* |
| *Cbx7* | *Dmd* | *Bai3* | *Tbx4* |
| *Cryab* | *Dysf* | *Blvrb* | *Tbx5* |
| *Dkk2* | *Enpp3* | *Chl1* | *Tnnt2* |
| *Dmrt2* | *Epha4* | *Chodl* |  |
| *Dok6* | *Evx2* | *Clgn* |  |
| *Dpp4* | *Fat3* | *Clvs2* |  |
| *Fabp7* | *Fgf13* | *Cntfr* |  |
| *Foxq1* | *Grem1* | *Cntn1* |  |
| *Fscn1* | *Hace1* | *Crlf1* |  |
| *Gbx2* | *Hand1* | *Cryab* |  |
| *Gdf5* | *Hemgn* | *Csrp3* |  |
| *Gpc3* | *Hgf* | *Ctgf* |  |
| *Grem1* | *Igf1* | *Egfr* |  |
| *Gria2* | *Isl1* | *Epha3* |  |
| *Grpr* | *Jazf1* | *Ermap* |  |
| *Hand1* | *Kat2b* | *Extl1* |  |
| *Hnf1b* | *Lbx1* | *Fat3* |  |
| *Irx6* | *Map2* | *Fgf11* |  |
| *Isl1* | *Myl3* | *Fitm1* |  |
| *Lbx1* | *Myog* | *Foxa3* |  |
| *Lect1* | *Nrp2* | *Fzd9* |  |
| *Myl1* | *Ntng2* | *Glp1r* |  |
| *Myl3* | *Ntrk1* | *Gna15* |  |
| *Myl4* | *Pax1* | *Gpm6b* |  |
| *Myo7a* | *Pde7b* | *Gpr37* |  |
| *Myog* | *Pdzd2* | *Gria1* |  |
| *Myom1* | *Plcb1* | *Hand1* |  |
| *Ncf2* | *Plek* | *Hcn3* |  |
| *Nrsn1* | *Pon2* | *Hdac9* |  |
| *Pax1* | *Rhag* | *Hic1* |  |
| *Pcdh8* | *Rxfp1* | *Hid1* |  |
| *Prox1* | *Sall1* | *Hsf4* |  |
| *Rgs5* | *Scn1b* | *Ihh* |  |
| *Rspo4* | *Scn3a* | *Inhba* |  |
| *S100g* | *Sntb1* | *Insc* |  |
| *Sfrp5* | *Spta1* | *Isl1* |  |
| *Sim2* | *Tbx4* | *Islr* |  |
| *Smpx* | *Tbx5* | *Kcns1* |  |
| *Smyd1* | *Timp3* | *Kera* |  |
| *Socs2* | *Tlr4* | *Kifc2* |  |
| *Sox10* | *Tnnc1* | *Klf4* |  |
| *Spi1* | *Tnnt2* | *Ldb3* |  |
| *Syn3* | *Trex1* | *Lmod2* |  |
| *Tbx4* | *Wnt5a* | *Lrp2* |  |
| *Tbx5* | *Zic3* | *Mafb* |  |
| *Tcf21* | *Zic5* | *Matn4* |  |
| *Tlr4* | *Zmat4* | *Mef2c* |  |
| *Tnnc1* |  | *Mmp19* |  |
| *Tnnc2* |  | *Moxd1* |  |
| *Tnnt2* |  | *Mpeg1* |  |
| *Wif1* |  | *Mtus2* |  |
| *Wnt2* |  | *Myf6* |  |
| *Zic1* |  | *Myl4* |  |
|  |  | *Myo1f* |  |
|  |  | *Myog* |  |
|  |  | *Myom1* |  |
|  |  | *Myoz2* |  |
|  |  | *Negr1* |  |
|  |  | *Nfat5* |  |
|  |  | *Ntrk1* |  |
|  |  | *Nxph1* |  |
|  |  | *Otor* |  |
|  |  | *Pak7* |  |
|  |  | *Parm1* |  |
|  |  | *Pax1* |  |
|  |  | *Pde1c* |  |
|  |  | *Pde3a* |  |
|  |  | *Pkdcc* |  |
|  |  | *Plcl1* |  |
|  |  | *Postn* |  |
|  |  | *Prox1* |  |
|  |  | *Ptprr* |  |
|  |  | *Pygm* |  |
|  |  | *Rab17* |  |
|  |  | *Reln* |  |
|  |  | *Rerg* |  |
|  |  | *Rfx4* |  |
|  |  | *Rgs16* |  |
|  |  | *Rgs8* |  |
|  |  | *Rxfp1* |  |
|  |  | *Scel* |  |
|  |  | *Sfrp5* |  |
|  |  | *Sim2* |  |
|  |  | *Slit2* |  |
|  |  | *Smyd1* |  |
|  |  | *Sox2* |  |
|  |  | *Spon1* |  |
|  |  | *Stac* |  |
|  |  | *Susd5* |  |
|  |  | *Syt6* |  |
|  |  | *Tanc2* |  |
|  |  | *Tbx3* |  |
|  |  | *Tbx4* |  |
|  |  | *Tbx5* |  |
|  |  | *Tcf21* |  |
|  |  | *Tecrl* |  |
|  |  | *Tlr5* |  |
|  |  | *Tnik* |  |
|  |  | *Tnni1* |  |
|  |  | *Tnnt2* |  |
|  |  | *Tusc5* |  |
|  |  | *Unc5a* |  |
|  |  | *Upp1* |  |
|  |  | *Wnt2* |  |
|  |  | *Zfhx4* |  |
