# Supplementary material for: Transcriptomic insights into the genetic basis of mammalian limb diversity
Source: BMC Evol Biol. 2017 Mar 23;17:86. doi: 10.1186/s12862-017-0902-6 (PMC5364624; doi:10.1186/s12862-017-0902-6)
Supplement: Supplementary file 9 — Genes with known roles in limb development (as identified by DAVID) that exhibit greatly divergent expression (75th percentile and above) among the limbs of all species. (DOCX 16 kb) [file 12862_2017_902_MOESM9_ESM.docx]

Table S2. Genes with known roles in limb development (as identified by DAVID) that exhibit greatly divergent expression (75^th^ percentile and above) among the limbs of all species.

| **FL ridge** | **FL bud** | **FL paddle** | **HL ridge** | **HL bud** | **HL paddle** |
| --- | --- | --- | --- | --- | --- |
| *Ctnnb1* | *Hoxa13* | *Ctnnb1* | *Ctnnb1* | *Col2a1* | *Col2a1* |
| *Dync2h1* | *Med1* | *Fbn2* | *Fbn2* | *Ctnnb1* | *Ctnnb1* |
| *Evx2* | *Pbx2* | *Fgf8* | *Fgf8* | *Fbn2* | *Fbn2* |
| *Fbn2* | *Prrx2* | *Hoxa13* | *Fgfr1* | *Fgf8* | *Fgfr1* |
| *Fgf8* | *Ptch1* | *Lnp* | *Hoxd11* | *Fgfr1* | *Hoxa13* |
| *Hoxa13* | *Shh* | *Med1* | *Hoxd9* | *Hoxd11* | *Hoxd11* |
| *Lnp* | *Zbtb16* | *Rarb* | *Lef1* | *Hoxd13* | *Hoxd13* |
| *Med1* |  |  | *Lnp* | *Hoxd9* | *Ift52* |
| *Prrx2* |  |  | *Med1* | *Mecom* | *Mecom* |
| *Ptch1* |  |  | *Msx1* | *Med1* | *Med1* |
| *Rarb* |  |  | *Nr2f2* | *Msx1* | *Msx1* |
| *Zbtb16* |  |  | *Pbx1* | *Pbx1* | *Pitx1* |
|  |  |  | *Pitx1* | *Pitx1* | *Prrx1* |
|  |  |  | *Prrx1* | *Prrx1* | *Rarb* |
|  |  |  | *Rarb* | *Rarb* | *Shox2* |
|  |  |  | *Tbx4* | *Shox2* | *Tbx4* |
|  |  |  | *Twist1* | *Tbx4* | *Twist1* |
|  |  |  |  | *Twist1* | *Wnt5a* |
|  |  |  |  | *Wnt5a* |  |
