# Supplementary material for: Transcriptomic insights into the genetic basis of mammalian limb diversity
Source: BMC Evol Biol. 2017 Mar 23;17:86. doi: 10.1186/s12862-017-0902-6 (PMC5364624; doi:10.1186/s12862-017-0902-6)

**Table S7**: Alignment of bat reads to the *Myotis lucifugus* genome using TOPHAT. FL and HL correspond to forelimb and hindlimb.


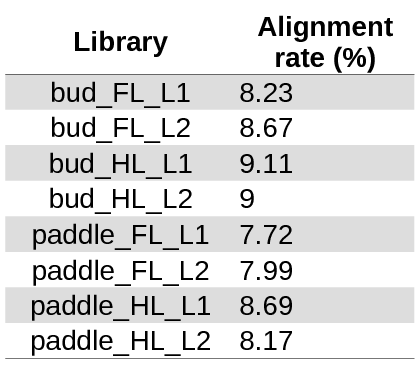

Supplement: Supplementary file 14 — Alignment of bat reads to the Myotis lucifugus genome using TOPHAT. FL and HL correspond to forelimb and hindlimb. (DOCX 29 kb) [file 12862_2017_902_MOESM14_ESM.docx]
