# Supplementary material for: Transcriptomic insights into the genetic basis of mammalian limb diversity
Source: BMC Evol Biol. 2017 Mar 23;17:86. doi: 10.1186/s12862-017-0902-6 (PMC5364624; doi:10.1186/s12862-017-0902-6)
Supplement: Supplementary file 15 — Embryo samples used for whole-mount in situ hybridization. (DOCX 15 kb) [file 12862_2017_902_MOESM15_ESM.docx]

|  | *Evx2* | *Hoxa13* | *Hoxd13* | *Hoxd12* |
| --- | --- | --- | --- | --- |
| Mus E9.5 | - | 1 | 1 | - |
| Mus E10.0 | 3 | 2 | 1 | - |
| Mus E10.5 | 4 | 2 | 3 | - |
| Mus E11.5 | 5 | 4 | 5 | - |
| Mus E12.5 | 3 | 3 | 2 | - |
| Bat CS13 | - | 1 | 2 | 1 |
| Bat CS14 | - | 1 | 2 | 1 |
| Bat CS15 | - | 2 | 1 | 1 |
| Op St 27 | 1 | 1 | 1 | 1 |
| Op St 28 | 3 | 1 | 2 | 2 |
| Op St 29 | 3 | 3 | 3 | 3 |
| Op St 31 | 3 | - | 2 | 2 |
| Op St 32 | 3 | 3 | 3 | 2 |

Table S6: Embryo samples used for whole mount *in situ* hybridization
