# Supplementary material for: Transcriptomic insights into the genetic basis of mammalian limb diversity
Source: BMC Evol Biol. 2017 Mar 23;17:86. doi: 10.1186/s12862-017-0902-6 (PMC5364624; doi:10.1186/s12862-017-0902-6)
Supplement: Supplementary file 16 — Accession Numbers & Primer Sequences For WISH. (DOCX 15 kb) [file 12862_2017_902_MOESM16_ESM.docx]

**Table S4**: Accession Numbers & Primer Sequences For WISH

| **Species** | **Gene** | **Forward Primer** | **Reverse Primer** | **Accession Number** |
| --- | --- | --- | --- | --- |
| *Carollia* | *Hoxa13* | ACGTGGTCTCTCATCCCTCA | TGGAGGAGCTTTCTCTCCCT | XM_006088760.1 |
| *Carollia* | *Hoxd12* | ATGTGTGAGCGCAGTCTCTACAG | TTAATATAGCGCCAGCGCCTGCT | XM_006083089.1 |
| *Carollia* | *Hoxd13* | CGGTATGGGCTTACAGCAGA | CTTTCGGATAGGTTCGTGGC | XM_006083259.1 |
| *Monodelphis* | *Evx2* | TATTTAGAGCCGCTCGCTGG | GGTAGCGTCTCACCTGATCG | XM_001368351 |
| *Monodelphis* | *Hoxa13* | GGAGTTCGCCTTCTACCACC | CTTGGGCTTTCGGGAGAACT | XM_001362439 |
| *Monodelphis* | *Hoxd12* | TCCAACCTGAGAGCGAATGG | AGTTCTGCGATCTGCTGCTT | XM_001368427.2 |
| *Monodelphis* | *Hoxd13* | ACATGGATGGACTTCGAGCG | AGGGTTGGTATAGCCCTGGT | XM_001368389 |
