# Supplementary material for: Transcriptomic insights into the genetic basis of mammalian limb diversity
Source: BMC Evol Biol. 2017 Mar 23;17:86. doi: 10.1186/s12862-017-0902-6 (PMC5364624; doi:10.1186/s12862-017-0902-6)
Supplement: Supplementary file 17 — Genes with known roles in limb development that exhibit greatly divergent expression in the fore- and hind limbs of a single species and among the limbs of all species. (DOCX 20 kb) [file 12862_2017_902_MOESM17_ESM.docx]

Table S3. Genes with known roles in limb development that exhibit greatly divergent expression in the fore- and hind limbs of a single species and among the limbs of all species. DAVID did not identify any known limb genes in the opossum forelimb ridge, bat forelimb ridge, pig hind limb ridge, mouse forelimb bud, bat forelimb bud, bat hind limb bud, opossum forelimb paddle, bat forelimb paddle, pig forelimb paddle, and bat hind limb paddle gene lists.

| RIDGE |  | BUD |  | PADDLE |  |
| --- | --- | --- | --- | --- | --- |
| Mouse forelimb | *Evx2* | Opossum forelimb | *Hoxa13* | Mouse forelimb | *Fgf8* |
|  | *Hoxa13* |  | *Prrx2* |  | *Lnp* |
|  | *Prrx2* |  | *Shh* |  | *Rarb* |
|  | *Rarb* |  | *Zbtb16* | Mouse hind limb | *Tbx4* |
|  | *Zbtb16* | Pig forelimb | *Hoxa13* |  | *Col2a1* |
|  | *Six2* |  | *Ptch1* |  | *Hoxd11* |
| Pig forelimb | *Evx2* |  | *Shh* |  | *Pitx1* |
|  | *Hoxa13* |  | *Zbtb16* |  | *Rarb* |
|  | *Ptch1* | Bat hind limb | *Tbx4* |  | *Wnt5a* |
| Bat hind limb | *Tbx4* |  | *Pitx1* | Opossum hind limb | *Tbx4* |
|  | *Prrx1* |  | *Pbx1* |  | *Col2a1* |
|  | *Pitx1* |  | *Wnt5a* |  | *Mecom* |
| Mouse hind limb | *Tbx4* | Mouse hind limb | *Tbx4* |  | *Hoxa13* |
|  | *Hoxd9* |  | *Ctnnb1* |  | *Hoxd11* |
|  | *Pitx1* |  | *Mecom* |  | *Hoxd13* |
|  | *Rarb* |  | *Hoxd9* |  | *Msx1* |
| Opossum hind limb | *Tbx4* |  | *Pitx1* |  | *Pitx1* |
|  | *Hoxd9* |  | *Shox2* |  | *Rarb* |
|  | *Lef1* |  | *Wnt5a* |  | *Wnt5a* |
|  | *Pitx1* | Opossum hind limb | *Tbx4* | Pig hind limb | *Tbx4* |
|  |  |  | *Col2a1* |  | *Col2a1* |
|  |  |  | *Mecom* |  | *Fbn2* |
|  |  |  | *Hoxd13* |  | *Hoxd13* |
|  |  |  | *Msx1* |  | *Rarb* |
|  |  |  | *Pitx1* |  | *Wnt5a* |
|  |  |  | *Wnt5a* |  |  |
|  |  | Pig hind limb | *Tbx4* |  |  |
|  |  |  | *Mecom* |  |  |
|  |  |  | *Fbn2* |  |  |
|  |  |  | *Hoxd13* |  |  |
|  |  |  | *Msx1* |  |  |
|  |  |  | *Rarb* |  |  |
|  |  |  | *Wnt5a* |  |  |
