# Supplementary material for: Transcriptomic insights into the genetic basis of mammalian limb diversity
Source: BMC Evol Biol. 2017 Mar 23;17:86. doi: 10.1186/s12862-017-0902-6 (PMC5364624; doi:10.1186/s12862-017-0902-6)
Supplement: Supplementary file 18 — Samples Used in Final Analysis. (DOCX 21 kb) [file 12862_2017_902_MOESM18_ESM.docx]

**Table S5**: Samples Used in Final Analysis

| **Limb** | **Library** | **Number of Reads** | **Alignment rate (%)** |
| --- | --- | --- | --- |
| FL Ridge | mouse_FL_W2_rep1 | 49774923 | 96.979 |
| FL Ridge | mouse_FL_W2_rep2 | 19601790 | 98.6523 |
| FL Ridge | mouse_FL_W2_rep3 | 23384986 | 97.4812 |
| FL Bud | mouse_FL_W3_4_rep1 | 24653734 | 98.3271 |
| FL Bud | mouse_FL_W3_4_rep2 | 25975513 | 97.8826 |
| FL Paddle | mouse_FL_W6_rep1 | 28252740 | 98.7349 |
| FL Paddle | mouse_FL_W6_rep2 | 61298693 | 98.6541 |
| FL Paddle | mouse_FL_W6_rep3 | 59415007 | 98.37 |
| HL Ridge | mouse_HL_W2_rep1 | 33052064 | 98.7322 |
| HL Ridge | mouse_HL_W2_rep2 | 37934065 | 98.3087 |
| HL Bud | mouse_HL_W3_4_rep1 | 35658149 | 98.9685 |
| HL Bud | mouse_HL_W3_4_rep2 | 20479039 | 98.2345 |
| HL Bud | mouse_HL_W3_4_rep3 | 54699003 | 98.7093 |
| HL Paddle | mouse_HL_W6_rep1 | 31215028 | 98.3112 |
| HL Paddle | mouse_HL_W6_rep2 | 20790601 | 98.0267 |
| HL Paddle | mouse_HL_W6_rep3 | 32427024 | 98.6401 |
| HL Paddle | mouse_HL_W6_rep4 | 22232610 | 98.7156 |
| FL Ridge | pig_FL_20_rep1 | 26057023 | 91.928 |
| FL Bud | pig_FL_22_rep1 | 56887620 | 92.4877 |
| FL Bud | pig_FL_22_rep2 | 51403715 | 92.4981 |
| FL Bud | pig_FL_22_rep3 | 31356905 | 91.307 |
| FL Paddle | pig_FL_26_rep1 | 42897988 | 91.7696 |
| FL Paddle | pig_FL_26_rep2 | 34392209 | 91.807 |
| FL Paddle | pig_FL_26_rep3 | 35755009 | 91.1923 |
| FL Paddle | pig_FL_26_rep4 | 34401744 | 90.6529 |
| HL Ridge | pig_HL_20_rep1 | 49547942 | 87.476 |
| HL Ridge | pig_HL_20_rep2 | 63675110 | 91.8537 |
| HL Bud | pig_HL_22_rep1 | 31010728 | 91.3773 |
| HL Bud | pig_HL_22_rep2 | 42887443 | 93.0277 |
| HL Bud | pig_HL_22_rep3 | 19272164 | 91.1548 |
| HL Paddle | pig_HL_26_rep1 | 34989242 | 91.9365 |
| HL Paddle | pig_HL_26_rep2 | 36956825 | 92.0233 |
| HL Paddle | pig_HL_26_rep3 | 22985345 | 91.7207 |
| HL Paddle | pig_HL_26_rep4 | 39219180 | 91.9551 |
| FL Ridge | opossum_FL_27_1 | 54327662 | 93.492 |
| FL Ridge | opossum_FL_27_2 | 32010769 | 93.0409 |
| FL Ridge | opossum_FL_27_3 | 27488110 | 91.3644 |
| FL Bud | opossum_FL_28_1 | 58632410 | 90.9821 |
| FL Bud | opossum_FL_28_2 | 37776593 | 90.8274 |
| FL Bud | opossum_FL_28_3 | 31252290 | 87.0133 |
| FL Bud | opossum_FL_28_4 | 37183598 | 90.8772 |
| FL Bud | opossum_FL_28_5 | 19568207 | 91.3338 |
| FL Bud | opossum_FL_28_6 | 77530354 | 86.6846 |
| FL Paddle | opossum_FL_29_1 | 58665501 | 92.0155 |
| FL Paddle | opossum_FL_29_2 | 39145942 | 90.2657 |
| FL Paddle | opossum_FL_29_3 | 77194292 | 92.2021 |
| HL Ridge | opossum_HL_30_1 | 40868746 | 91.8085 |
| HL Ridge | opossum_HL_30_2 | 27990650 | 91.7669 |
| HL Ridge | opossum_HL_30_3 | 26649172 | 91.0475 |
| HL Bud | opossum_HL_31_1 | 27286008 | 92.0572 |
| HL Bud | opossum_HL_31_2 | 36194500 | 91.8843 |
| HL Bud | opossum_HL_31_3 | 53668769 | 92.3268 |
| HL Bud | opossum_HL_31_4 | 48954912 | 92.0553 |
| HL Bud | opossum_HL_31_5 | 68360943 | 92.1626 |
| HL Paddle | opossum_HL_32_1 | 14914988 | 91.8143 |
| HL Paddle | opossum_HL_32_2 | 34046140 | 91.3137 |
| HL Paddle | opossum_HL_32_3 | 36318003 | 92.3339 |
| FL Ridge | bat_FL_13_rep1 | 17916012 | 82.5077 |
| FL Ridge | bat_FL_13_rep2 | 39417628 | 81.859 |
| FL Ridge | bat_FL_13_rep3 | 37481659 | 80.7875 |
| FL Bud | bat_FL_14_rep1 | 44364335 | 80.1456 |
| FL Bud | bat_FL_14_rep2 | 39030095 | 78.6385 |
| FL Bud | bat_FL_14_rep3 | 29851584 | 80.4992 |
| FL Paddle | bat_FL_15_rep1 | 26539725 | 76.6049 |
| FL Paddle | bat_FL_15_rep2 | 23022666 | 77.2736 |
| HL Ridge | bat_HL_13_rep1 | 20774208 | 85.5907 |
| HL Ridge | bat_HL_13_rep2 | 30617563 | 83.8383 |
| HL Ridge | bat_HL_13_rep3 | 22463496 | 82.6644 |
| HL Bud | bat_HL_14_rep1 | 24628892 | 79.7932 |
| HL Bud | bat_HL_14_rep2 | 37554686 | 80.349 |
| HL Bud | bat_HL_14_rep3 | 84537765 | 80.1107 |
| HL Paddle | bat_HL_15_rep1 | 51702286 | 79.2226 |
| HL Paddle | bat_HL_15_rep2 | 23367600 | 79.245 |
